# Supplementary material for: TIMEPOINT, a phase 1 study combining MTL-CEBPA with pembrolizumab, supports the immunomodulatory effect of MTL-CEBPA in solid tumors
Source: Cell Rep Med. 2025 Mar 31;6(4):102041. doi: 10.1016/j.xcrm.2025.102041 (PMC12047497; doi:10.1016/j.xcrm.2025.102041)
Supplement: Document S1. Figures S1–S5 and Tables S1–S4 [file mmc1.pdf]

Cell Reports Medicine, Volume *6*

Supplemental information

TIMEPOINT, a phase 1 study combining MTL-CEBPA with pembrolizumab, supports the immunomodulatory effect of MTL-CEBPA in solid tumors

Ruth Plummer, Mikael H. Sodergren, Rose Hodgson, Bríd M. Ryan, Nina Raulf, Joanna P. Nicholls, Vikash Reebye, Jon Voutila, Laura Sinigaglia, Tim Meyer, David J. Pinato, Debashis Sarker, Bristi Basu, Sarah Blagden, Natalie Cook, Thomas R. Jeffrey Evans, Jeffrey Yachnin, Cheng E. Chee, Daneng Li, Anthony El-Khoueiry, Maria Diab, Kai-Wen Huang, Madhava Pai, Duncan Spalding, Thomas Talbot, Marcus S. Noel, Bridget Keenan, Devalingam Mahalingam, Min-Sun Song, Mélanie Grosso, Denis Arnaud, Aurelie Auguste, Dimitris Zacharoulis, Jan Storkholm, Iain McNeish, Robert Habib, John J. Rossi, and Nagy A. Habib

A

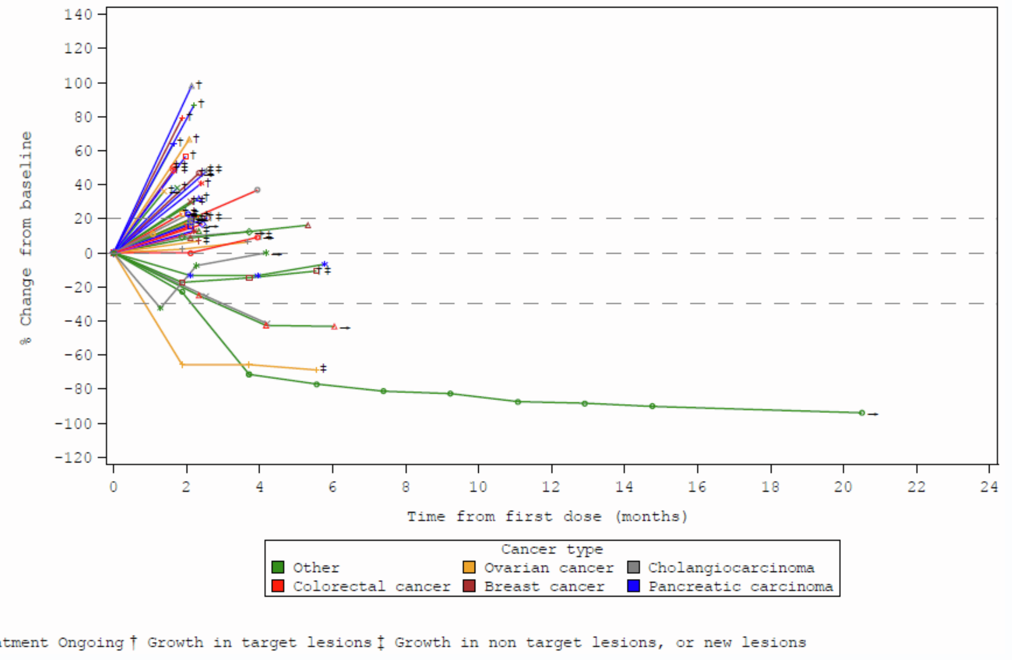

Supplementary Figure 1. Best objective tumour response by patient across treatment groups in TIMEPOINT clinical trial.

A. Spider plot waterfall plot illustrating patient best objective tumour response across treatment groups in Phase 1a and Phase 1b. Note: an i prefix represents a response evaluated according to the iRECIST or irRECIST criteria. Patients with no post-baseline RECIST assessments are excluded from this figure. iRECIST=immune RECIST; irRECIST=immune-related RECIST; PD=progressive disease; PR=partial response; SD=stable disease; UPD=unconfirmed progressive disease. Each line represents one patient. Related to Figure 1.

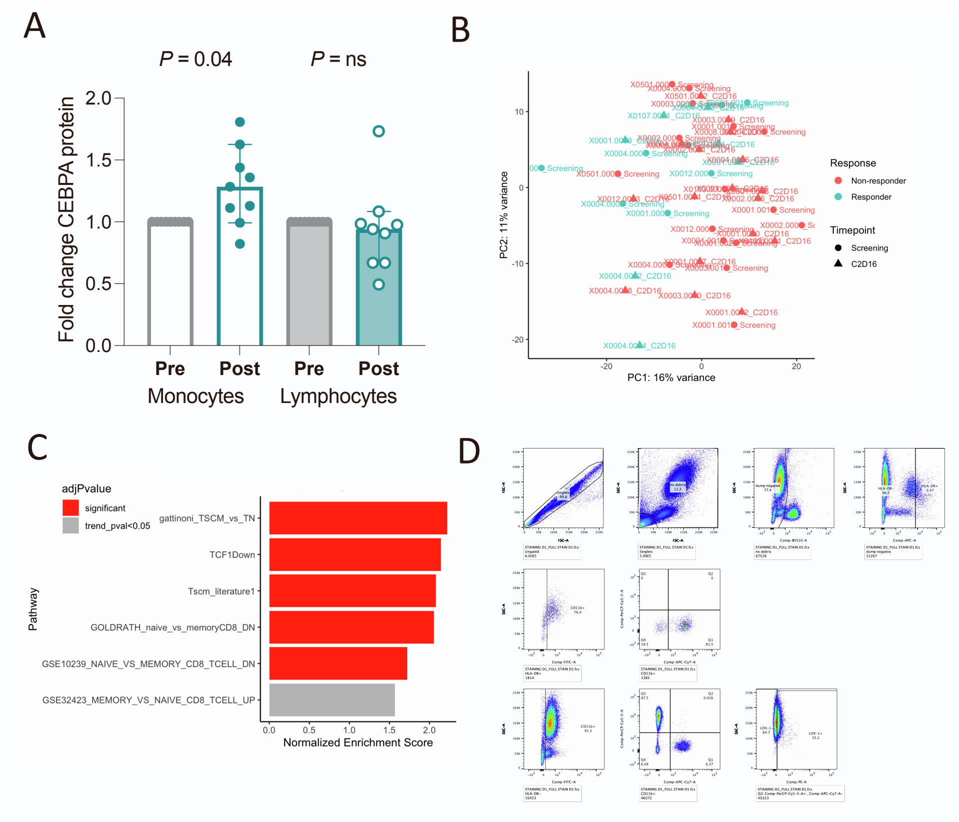

Supplemental figure 2. Pharmacodynamic changes seen in patient tumour and peripheral blood samples post-treatment with MTL-CEBPA in combination with pembrolizumab.

A. Detection of CEBPA protein in patient monocyte or lymphocyte fraction enriched from peripheral blood by density gradient centrifugation, pre (C1D1) and post treatment (C1 D2) with MTL-CEBPA. Each point represents one patient. Bar is at median with 95% confidence intervals as error bars. Significance was determined by one sample Wilcoxon test with hypothetical median value of 1, P values are unadjusted. B. PCAplot of 48 patient samples of Nanostring transcriptomic data analysed with DESeq and VST normalization and stabilization for batch effects using pre-assigned housekeepers. Note sample 0107-0001 patient had low absolute counts but clusters normally with DESeq2 analysis. Response refers to clinical response at C2 D22 determined by site. C. FGSEA analysis of differentially expressed gene pathways between pre- and post-treatment timepoints across all patients, 14 using T stem cell memory gene sets identified or curated from literature (Supplementary table 8). Pathways in red reached significance (P adjust < 0.05), grey pathways were significant pre-adjustment for multiple testing (P <0.05). D. Representative flow cytometry plots of full gating path example of PMN-MDSCs from peripheral blood. Gating is based on FMOs for each marker. Related to Figure 2.

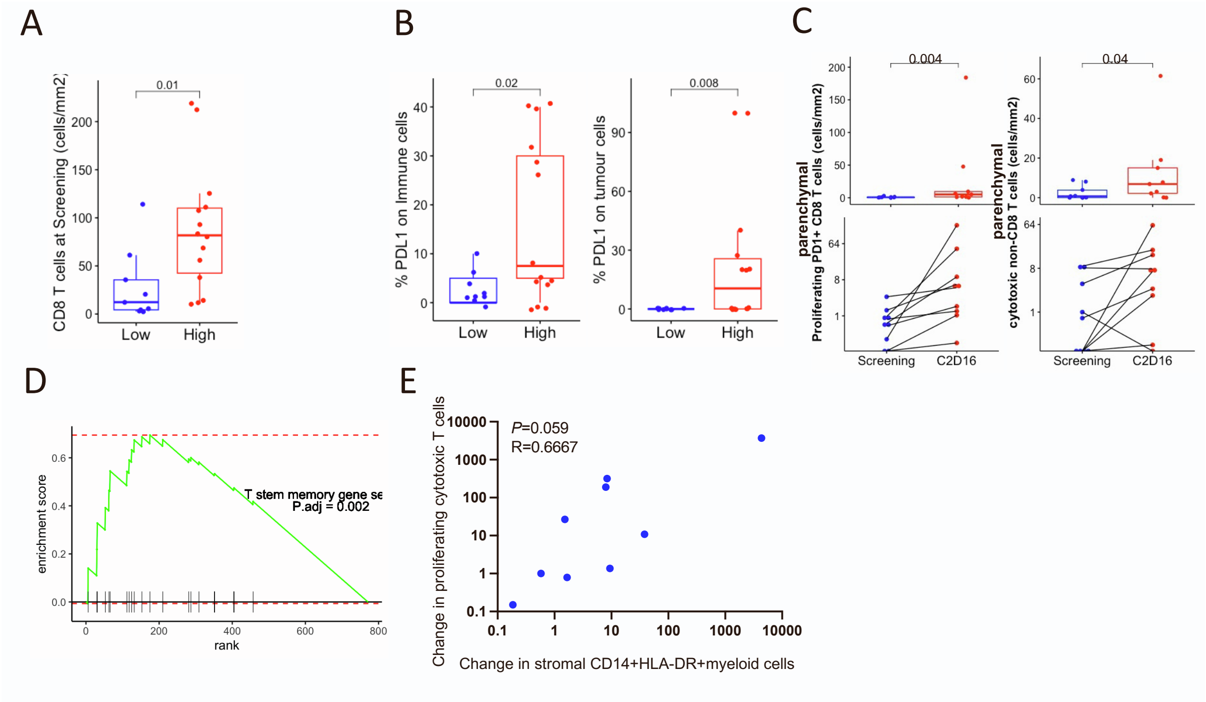

Supplementary figure 3. Patient tumour biopsies have different levels of immune infiltration before treatment, with patients that have lower numbers of immune cells at baseline demonstrating increased intratumoural immune infiltration following treatment with MTL-CEBPA in combination with pembrolizumab.

A. IHC quantification of CD3+CD8+ T cells at pre-treatment (Screening) between of 9 tumour biopsies with an immune desert TME (IS21 < 10) and 14 patient biopsies with an immune-inflamed TME (IS21 > 10). The threshold of 10 for IS21 was arbitrarily chosen as it is the midpoint of the assay readout, which gives values between 0-20. P value is determined by two-sided Wilcoxon test. B. IHC quantification of PDL1 presence on immune cells (IC: left) or tumour cells (TPS: right) at pre-treatment (Screening) between of 9 tumour biopsies with an immune desert TME (IS21 < 10) and 14 patient biopsies with an immune-inflamed TME (IS21 > 10). P value is determined by two-sided Wilcoxon test. C. IHC quantification of parenchymal CD3+ CD8+KI67+GrzB+PD1+ T cells (left) and parenchymal CD3+CD8-GZMB+ T cells (right) between pre- (Screening) and post-treatment (C2D16) of 9 paired biopsies with a immune desert TME (IS21 < 10). P value is determined by Paired Two-sided Wilcoxon test. For A, B and C, the box plots show the data distribution, where the center line denotes the median, the box edges show the interquartile range each point is one patient. D. Gene set enrichment plot of T stem memory gene set, "GSE10239_NAIVE_VS_MEMORY_CD8_TCELL_DN". Significance was determined by fgsea and adjusted for multiple testing based on 14 gene sets tested (Supplementary table 8). E. Pearson correlation of change in stromal CD14+HLA-DR+ myeloid cells from

Screening to C2D16 with change in proliferating cytotoxic CD8 T cells in patients that had cold TME at baseline (N=9). Related to figure 3.

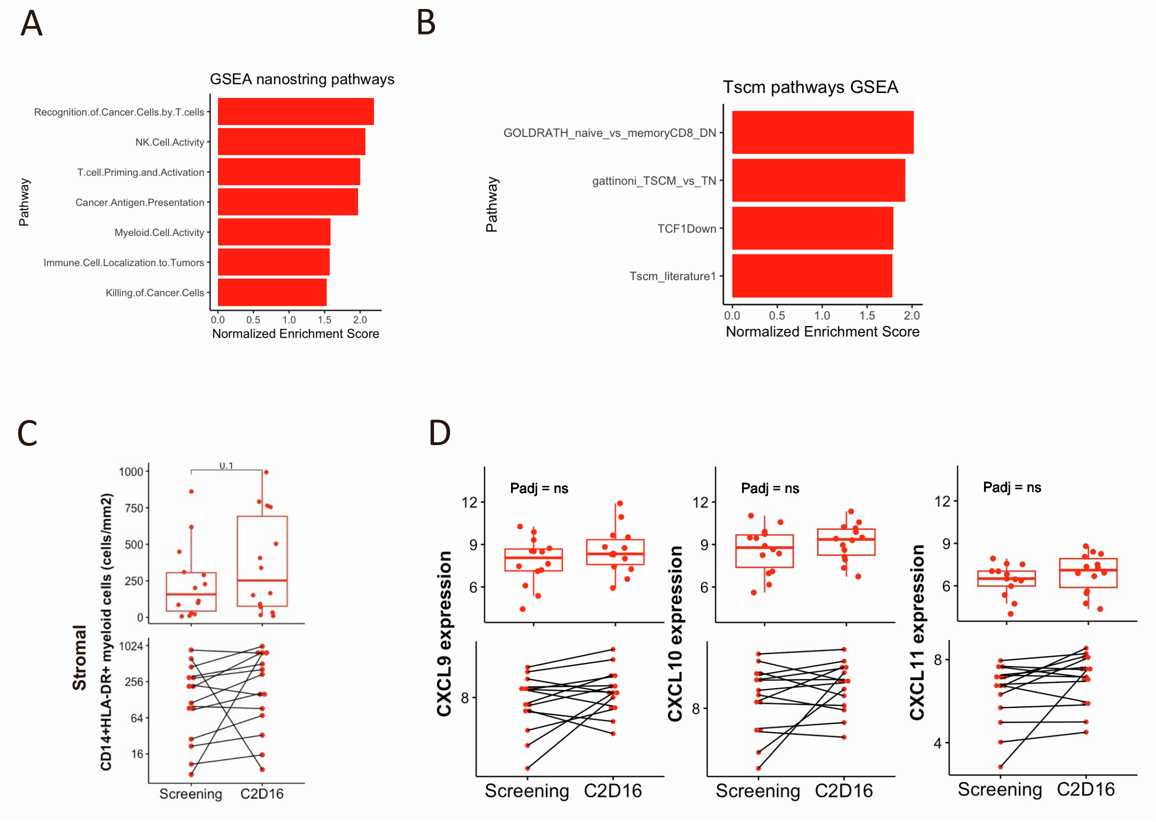

Supplementary figure 4. Patients with high levels of immune activity at baseline demonstrate modest immune infiltration following combination treatment with MTL-CEBPA in combination with pembrolizumab.

A. FGSEA analysis of differentially expressed genes between pre- (Screening) and post-treatment (C2D16) of 14 paired biopsies with an immune inflamed TME (IS21 > 10), using supplied Nanostring pathways of the IO Pan-cancer panel or B. T stem cell memory pathways. Pathways in red reached significance with adjustment for multiple testing for 13 (A) or 14 (B) gene sets (P adjust < 0.05). C. IHC quantification of stromal CD11b+CD14+CD15-HLA-DR+ cells between pre- (Screening) and post-treatment (C2D16) in the stroma of 14 paired biopsies with an immune inflamed TME (IS21 > 10). P value is determined by Paired Two-sided Wilcoxon test. D. Quantification of CXCL genes post-treatment in immune inflamed tumours by nanostring. Data are VST values normalised for batch with housekeepers, with significance determined by DESeq2 on raw data. The box plots in C and D show the data distribution, where the center line denotes the median, the box edges show the interquartile range and each point represents one patient. Related to Figure 3.

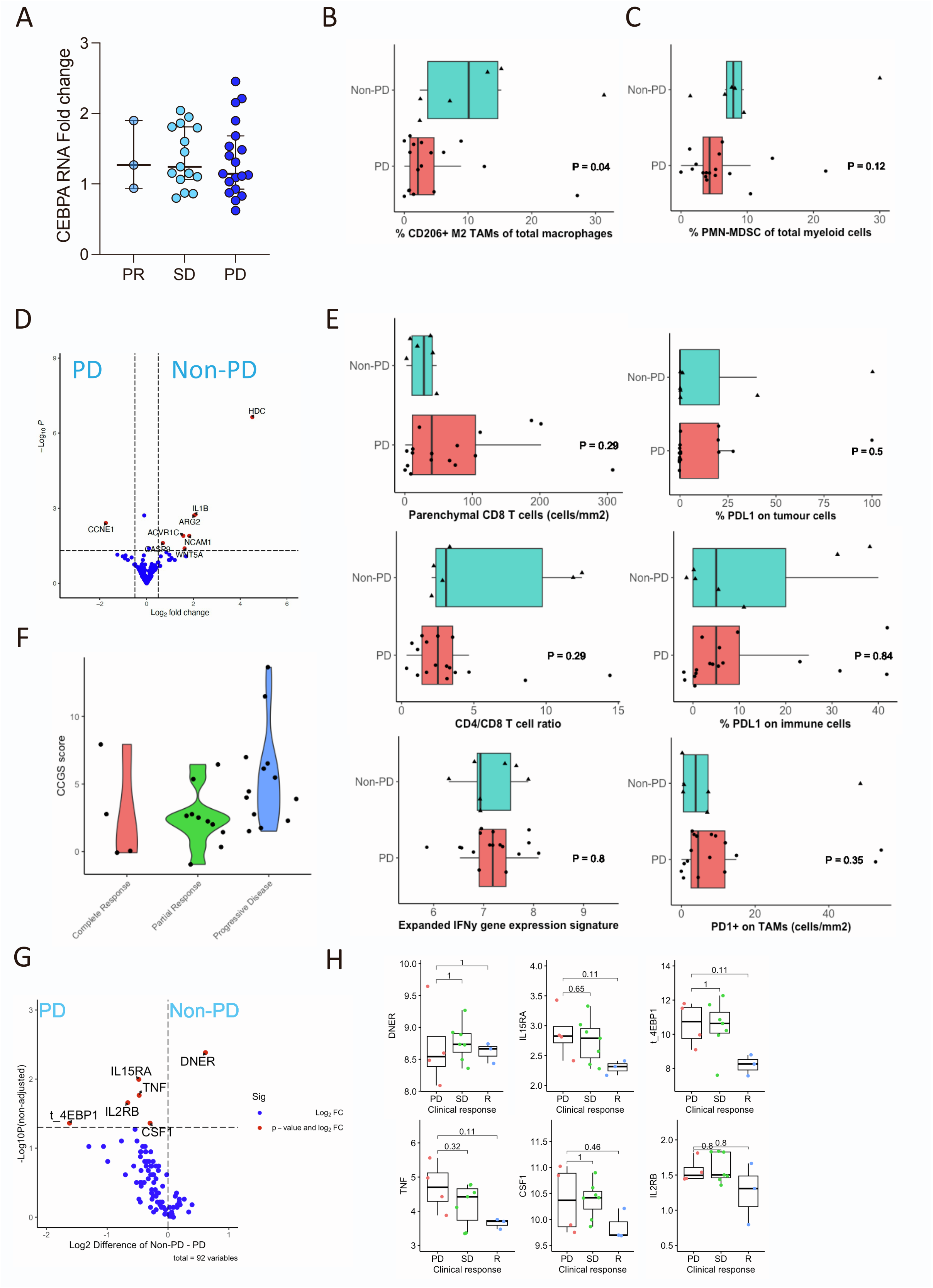

Supplementary Figure 5. Patients that have signals associated with myeloid-derived immunosuppression pre-treatment are more likely to demonstrate disease stabilisation following treatment with MTL-CEBPA in combination with pembrolizumab.

A. Fold change of CEBPA RNA at C1 D2 compared to pre-treatment, C1-D1 in TIMEPOINT in patients with PR, SD and PD. Response refers to clinical response at C2 D22 determined by site. Bar is at median with 95% confidence interval error bars, each point is one patient. Significance was tested with a Kruskal-Wallis test, with no significance reached at a P value of P = 0.05. B. Visualisation of CD11b+CD68+CD64-CD206+CD163- cells (CD206+ M2 TAMs) as a proportion of total macrophages (CD11b+CD68+ cells) between patients with PD and non-PD. C. Visualisation of CD11b+CD15+CD14-HLA-DR-LOX1+ (PMN-MDSC) cells as a proportion of total myeloid cells (CD11b+ cells) between patients with PD and non-PD. For B and C, each point is one patient, significance values were determined with a Two-sided Wilcoxon test. E. Visualisation of previously identified pembrolizumab monotherapy biomarkers between patients with PD and non-PD. Significance was determine using Two-sided Wilcoxon tests for each plot independently without adjustment for multiple testing. F. CCGS determined by RNASeq of pre-treatment gene expression in pembrolizumab treated melanoma patients from publicly available dataset, GSE78220, each point is one patient. G. Volcano plot of OLINK serum analysis of circulating proteins using Inflammation panel of patient serum at baseline (pre-treatment) in TIMEPOINT between patients with different responses. Significance was determined with an unpaired two-tailed Wilcoxon test, using patients with PD as the control group. P values are non-adjusted. All genes are labelled. Each point is one patient. For B-E and G, response refers to clinical response at C2 D22 determined by site. H. OLINK serum analysis of circulating proteins using Inflammation panel of patient serum at baseline (pre-treatment) in OUTREACH clinical trial as NPX values. Response refers to PD or non-PD clinical response determined by RECIST. Significant was determined with an unpaired two-tailed Wilcoxon. P values are non-adjusted. The box plots in B, C, E, and H show the data distribution, where the center line denotes the median, the box edges show the interquartile range and each point is one patient. Related to Figures 4 and 5.

Supplementary tables

Supplementary Table 1. Study population analysis sets by tumour type recruited in TIMEPOINT clinical trial.

EFR=evaluable for response; PKAS=pharmacokinetic analysis set; SAF=safety analysis set.

'Other' category contains the following cancer types: epithelioid mesothelioma, thymic cancer metastatic, hepatocellular carcinoma, eccrine carcinoma, adenocarcinoma, lung neoplasm malignant (term amended to mesothelioma after the data cut-off for this interim analysis), extrahepatic cholangiocarcinoma (in 2 patients), neuroendocrine tumour, leiomyosarcoma, malignant peritoneal neoplasm, anal squamous cell carcinoma, and mesothelioma. Related to Figure 1, table 1 and Supplementary figure 1.

**Cancer type**	**Breast cancer**	**Intrahepatic Cholangiocarcinoma**	**Colorectal cancer**	**Ovarian cancer**	**Pancreatic carcinoma**	**Other**	**Overall**
**Patient number**	**(N=4)**	**(N=7)**	**(N=9)**	**(N=8)**	**(N=9)**	**(N=13)**	**(N=50)**
**SAF**	4 (100.0)	7 (100.0)	9 (100.0)	8 (100.0)	9 (100.0)	13 (100.0)	50 100.0)
**PKAS**	1 (25.0)	1 (14.3)	1 (11.1)	4 (50.0)	3 (33.3)	6 (46.2)	16 (32.0)
**EFR**	3 (75.0)	5 (71.4)	6 (66.7)	7 (87.5)	8 (88.9)	11 (84.6)	40 (80.0)

Supplementary Table 2: Patient demographics at baseline in TIMEPOINT clinical trial.

QW=dosing frequency is once weekly, BMI= body mass index, BSA = body surface area, ECOG= Eastern Cooperative Oncology Group score. Related to Figure 1, table 1 and Supplementary figure 1.

	**Phase 1a**	**Phase 1a**	**Phase 1a**	**Phase 1b**	**MTL-CEBPA**
	**MTL-CEBPA**	**MTL-CEBPA**	**MTL-CEBPA**	**MTL-CEBPA**	**overall**
	**70 mg/m^2 QW**	**98 mg/m^2 QW**	**130 mg/m^2 QW**	**130 mg/m^2 QW**	**(N=50)**
	**(N=4)**	**(N=3)**	**(N=3)**	**(N=40)**	
**Age at time of informed consent (years)**					
Median (min;max)	44.5 (33;56)	56.0 (21;72)	48.0 (40;60)	62.5 (25;80)	58.5 (21;80)
**Gender**					
Female, n (%)	3 (75.0)	2 (66.7)	2 (66.7)	26 (65.0)	33 (66.0)
Male, n (%)	1 (25.0)	1 (33.3)	1 (33.3)	14 (35.0)	17 (34.0)
**Race**					
White/Caucasian, n (%)	4 (100.0)	1 (33.3)	2 (66.7)	30 (75.0)	37 (74.0)
Asian or Asian British, n (%)	0	2 (66.7)	1 (33.3)	5 (12.5)	8 (16.0)
Black or Black British, n (%)	0	0	0	2 (5.0)	2 (4.0)
Mixed	0	0	0	2 (5.0)	2 (4.0)
Other	0	0	0	1 (2.5)	1 (2.0)
**Baseline BMI (kg/m^2)**					
Mean ± StD	23.6 ± 3.2	24.4 ± 9.6	23.4 ± 3.5	25.2 ± 4.8	24.9 ± 4.8
Median (min;max)	22.9 (20.4;28.0)	19.4 (18.3;35.4)	22.0 (20.7;27.4)	24.4 (14.2;38.2)	24.0 (14.2;38.2)
**Baseline BSA (kg/m^2)**					
Median (min;max)	1.7 (1.6;1.7)	1.4 (1.3;1.8)	1.8 (1.76;1.78)	1.8 (1.4;2.2)	1.8 (1.3;2.2)
**ECOG status at baseline** Median (min;max)					
0, n (%)	3 (75.0)	2 (66.7)	1 (33.3)	17 (42.5)	23 (46.0)
1, n (%)	1 (25.0)	1 (33.3)	2 (66.7)	23 (57.5)	27 (54.0)

**Prior lines of chemotherapy**					
0	0	1 (33.3)	0	0	1 (2.0)
1	0	1 (33.3)	1 (33.3)	4 (10.0)	6 (12.0)
2	1 (25.0)	1 (33.3)	1 (33.3)	12 (30.0)	15 (30.0)
3	1 (25.0)	0	0	11 (27.5)	12 (24.0)
4	0	0	1 (33.3)	4 (10.0)	5 (10.0)
5	1 (25.0)	0	0	6 (15.0)	7 (14.0)
6	1 (25.0)	0	0	3 (7.5)	4 (8.0)

Supplementary table 3: Best objective tumour response across Treatment groups in Phase 1a and Phase 1b of TIMEPOINT clinical trial.

Note: an i prefix represents a response evaluated according to the iRECIST or irRECIST criteria. iRECIST=immune RECIST; irRECIST=immune-related RECIST; mRECIST=modified RECIST; PD=progressive disease; PR=partial response; QW=once a week; RECIST=Response Evaluation Criteria in Solid Tumours; SD=stable disease; trRECIST=tumour response RECIST; UPD=unconfirmed progressive disease. Related to Figure 1, table 1 and Supplementary figure 1.

	**Breast cancer**	**Intrahepatic Cholangiocarcinoma**	**Colorectal cancer**	**Ovarian cancer**	**Pancreatic carcinoma**	**Other**	**Overall**
	**(N=3)**	**(N=5)**	**(N=6)**	**(N=7)**	**(N=8)**	**(N=11)**	**(N=40)**
**Responder**	0	1 (20.0)	0	1 (14.3)	0	2 (18.2)	4 (10.0)
PR	0	1 (20.0)	0	1 (14.3)	0	2 (18.2)	4 (10.0)
PR (RECIST 1.1)	0	1 (20.0)	0	1 (14.3)	0	0	2 (5.0)
PR (mRECIST)	0	0	0	0	0	1 (9.1)	1 (2.5)
iPR (iRECIST)	0	0	0	0	0	1 (9.1)	1 (2.5)
**Non-responder**	3 (100.0)	4 (80.0)	6 (100.0)	6 (85.7)	8 (100.0)	9 (81.8)	36 (90.0)
SD (RECIST 1.1)	0	3 (60.0)	1 (16.7)	3 (42.9)	0	3 (27.3)	10 (25.0)
iSD (trRECIST)	0	0	1 (16.7)	0	0	0	1 (2.5)
PD (RECIST 1.1)	3 (100.0)	1 (20.0)	3 (50.0)	3 (42.9)	7 (87.5)	6 (54.5)	23 (57.5)
iUPD (iRECIST)	0	0	1 (16.7)	0	1 (12.5)	0	2 (5)

Supplementary table 4. Patient information for those with tissues analysed by biological assays for exploratory analysis. * For this patient, only pre-treatment tumour biopsy sample was available. Related to figures 2-5 and supplemental figures 2-5.

Patient ID	Tumourtype/Aetiology	IHC T cells	IHC myeloid	IHC PDL1	Nanostring	PDL1	OLINK
0001.0009	EccrineAdenocarcinoma	x	x	x	x	x	x
0001.0011	Oesophago-gastricAdenocarcinoma	x	x	x	x	x	x
0001.0012	OvarianSerousCarcinoma	x	x	x	x	x	x
0001.0015	Recto-sigmoid	x	x	x	x	x	x
0001.0016	Ovarian	x	x	x	x	x	x
0001.0017	ExtrahepaticCholangiocarcinoma	x	x	x	x	x	x
0001.0020	Colon	x	x	x	x	x	x
0002.0001	Pancreatic	x	x	x	x	x	
0002.0006	BreastCancer(TripleNeg)	x	x	x	x	x	x
0003.0009	Ovarian(lowgradeserous)	x	x	x	x	x	x
0003.0010	Ovarian(HighgradeSerous)	x	x	x	x	x	x
0004.0002	FibrolamellarHCC	x	x	x	x	x	x
0004.0003	Colon	x	x	x	x	x	x
0004.0004	LungMetastaticAtypicalLung	x	x	x	x	x	x
0004.0005	Pancreatic	x	x	x	x	x	x
0004.0006	Recto-sigmoid	x	x	x	x	x	x
0004.0008	Sarcoma	x	x	x	x	x	
0008.0002	PancreaticAdenocarcinoma	x	x	x	x	x	x
0012.0001	IntrahepaticCholangiocarcinoma	x	x	x	x	x	x
0012.0003	IntrahepaticCholangiocarcinoma	x	x	x	x	x	x
0103.0001	Pancreatic	x	x	x	x	x	x
0107.0001	RectalCancer				x*		x
0501.0001	IntrahepaticCholangiocarcinoma	x	x	x	x	x	x

0501.0002	Breast	x	x	x	x	x	x
0001.0013	Ovarian						
0001.0014	Mesothelioma(Epitheloid)						
0001.0003	Breast(TripleNeg)						
0001.0004	Mesothelioma(Epitheloid)						x
0001.0005	Ovarian						x
0001.0006	Thymic						
0001.0007	Fibrolamellar						
0001.0018	IntrahepaticCholangiocarcinoma						x
0001.0019	Rectal						
0101.0002	Pancreatic						
0101.0004	IntrahepaticCholangiocarcinoma						x
0001.0021	Mesothelioma(Epitheloid)						
0005.0002	Ovarian(Peritoneal)Adenocarcinoma						
0010.0002	Pancreatic(Neuroendocrine)						
0002.0002	PancreaticMetastaticAdenocarcinoma						
0002.0003	BreastCancer						
0003.0002	Colorectal						
0003.0004	intrahepaticCholangiocarcinoma						
0003.0005	Ovarian						
0003.0006	ExtrahepaticCholangiocarcinoma						
0003.0008	PancreaticAdenocarcinoma						
0101.0006	Pancreatic						
0101.0007	ColonCancer						
0005.0001	Rectal						x
0103.0003	IntrahepaticCholangiocarcinoma						x
0501.0003	Mesothelioma(Peritoneal)						x
0101.0005	Rectal(anal)Cancer						

0201.000<br>1	Ovariancancer-highgradeserous							
