# Supplementary material for: TIMEPOINT, a phase 1 study combining MTL-CEBPA with pembrolizumab, supports the immunomodulatory effect of MTL-CEBPA in solid tumors
Source: Cell Rep Med. 2025 Mar 31;6(4):102041. doi: 10.1016/j.xcrm.2025.102041 (PMC12047497; doi:10.1016/j.xcrm.2025.102041)
Supplement: Document S2. Article plus supplemental information [file mmc6.pdf]

Cell Reports
Medicine

Article

TIMEPOINT, a phase 1 study combining MTL-CEBPA with pembrolizumab, supports the immunomodulatory effect of MTL-CEBPA in solid tumors

Graphical abstract

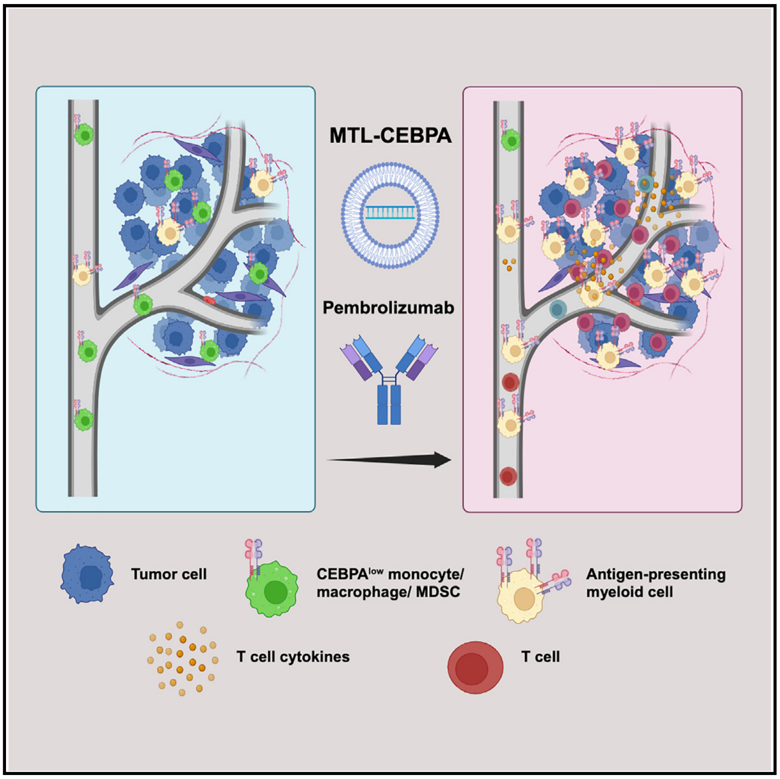

Highlights

- Combination MTL-CEBPA and pembrolizumab treatment was safe and well tolerated
- Treatment-associated switch of tumor environment from immune-desert to inflamed
- MTL-CEBPA-target myeloid immunosuppression predictive of disease stabilization

Authors

Ruth Plummer, Mikael H. Sodergren, Rose Hodgson, ..., Robert Habib, John J. Rossi, Nagy A. Habib

Correspondence

nagy.habib@imperial.ac.uk

In brief

Plummer et al. report positive immunomodulatory changes to the tumor microenvironment in patients with advanced solid cancers, after combination treatment with small activating RNA, MTL-CEBPA, and immune checkpoint inhibition. Patients with enrichment of myeloid-derived immunosuppression were more likely to demonstrate disease stabilization, supporting a role for MTL-CEBPA in TME immunomodulation.

Plummer et al., 2025, Cell Reports Medicine *6*, 102041
April 15, 2025 © 2025 The Authors. Published by Elsevier Inc.
https://doi.org/10.1016/j.xcrm.2025.102041

CellPress

Cell Reports Medicine

Cell Press
OPEN ACCESS

Article

TIMEPOINT, a phase 1 study combining MTL-CEBPA with pembrolizumab, supports the immunomodulatory effect of MTL-CEBPA in solid tumors

Ruth Plummer,[1,23] Mikael H. Sodergren,[2,23] Rose Hodgson,[3,23] Bríd M. Ryan,[3] Nina Raulf,[3] Joanna P. Nicholls,[2,3] Vikash Reebye,[2,3] Jon Voutila,[3] Laura Sinigaglia,[3] Tim Meyer,[4] David J. Pinato,[2,5] Debashis Sarker,[6] Bristi Basu,[7] Sarah Blagden,[8] Natalie Cook,[9] Thomas R. Jeffrey Evans,[10] Jeffrey Yachnin,[11] Cheng E. Chee,[12] Daneng Li,[13] Anthony El-Khoueiry,[14] Maria Diab,[15] Kai-Wen Huang,[16] Madhava Pai,[2] Duncan Spalding,[2] Thomas Talbot,[2] Marcus S. Noel,[17] Bridget Keenan,[18] Devalingam Mahalingam,[19] Min-Sun Song,[20] Mélanie Grosso,[21] Denis Arnaud,[21] Aurelie Auguste,[21] Dimitris Zacharoulis,[22] Jan Storkholm,[2] Iain McNeish,[2] Robert Habib,[3] John J. Rossi,[20] and Nagy A. Habib[2,3,24,25,*]

[1]The Northern Centre for Cancer Care, Freeman Hospital, NE7 7DN Newcastle, UK
[2]Department of Surgery & Cancer, Imperial College London, W12 0NN London, UK
[3]MiNA Therapeutics Ltd, W12 0BZ London, UK
[4]Research Department of Oncology, UCL Cancer Institute, University College London, WC1E 6DD London, UK
[5]Department of Translational Medicine (DIMET), Università Del Piemonte Orientale "A. Avogadro", Novara, Italy
[6]Department of Research Oncology, Guys Hospital, Kings College London, SE1 9RT London, UK
[7]University of Cambridge and Cambridge University Hospitals NHS Foundation Trust, CB2 0QQ Cambridge, UK
[8]Department of Oncology, Oxford University, Churchill Hospital, OX3 7LE Oxford, UK
[9]University of Manchester and The Christie NHS Foundation Trust, M20 4BX Manchester, UK
[10]The Beatson West of Scotland Cancer Centre, University of Glasgow, G12 0YN Glasgow, UK
[11]Centrum Kliniska Cancerstudier, Karolinska University Hospital, 17176 Stockholm, Sweden
[12]National University Hospital, National University Cancer Institute Singapore, Singapore 11928, Singapore
[13]Department of Medical Oncology & Therapeutics Research, City of Hope Comprehensive Cancer Center, Duarte, CA 91010, USA
[14]Norris Comprehensive Cancer Centre, Keck Medicine, University of Southern California, Los Angeles, CA 90089, USA
[15]Winship Cancer Institute, Emory University, Atlanta, GA 30322, USA
[16]National Taiwan University Hospital, Taipei, Taiwan
[17]Medstar Georgetown University Hospital, Washington, DC 20007, USA
[18]University of California San Francisco, San Francisco, CA 94143, USA
[19]Robert H Lurie Comprehensive Cancer Centre, Northwestern University, Chicago, IL 60611, USA
[20]Beckman Research Institute, City of Hope, CA, USA
[21]Veracyte, Marseilles, France
[22]Department of General Surgery, University Hospital of Larissa, Larissa, Greece
[23]These authors contributed equally
[24]Senior author
[25]Lead contact
*Correspondence: nagy.habib@imperial.ac.uk
https://doi.org/10.1016/j.xcrm.2025.102041

SUMMARY

Many patients with cancer do not benefit from currently approved immune checkpoint inhibitors (ICIs), suggesting that additional immunomodulation of the immunosuppressive tumor microenvironment (TME) is required. MTL-CCAAT enhancer-binding protein alpha (CEBPA) specifically upregulates the expression of the master myeloid transcription factor, CEBPA, relieving myeloid-driven immunosuppression. Here, we report the safety, tolerability, pharmacokinetics, and efficacy of MTL-CEBPA in combination with pembrolizumab in patients with advanced solid tumors that typically show ICI resistance. Multimodal exploratory analyses of paired patient biopsies demonstrate biological changes associated with the combination treatment of MTL-CEBPA and pembrolizumab, including increased infiltration of T cell and antigen-presenting cells supporting conversion from an immune-desert toward a more immune-inflamed TME. Patients with disease stabilization demonstrate reductions in immunosuppressive myeloid cells post treatment. Collectively, these data support a role for MTL-CEBPA in reducing immunosuppression in the TME. This study was registered at ClinicalTrials.gov (NCT04105335).

© 2025 The Authors. Published by Elsevier Inc.
This is an open access article under the CC BY license (http://creativecommons.org/licenses/by/4.0/).

INTRODUCTION

Immunotherapy in the form of checkpoint blockade has revolutionized systemic cancer therapy and for some cancers has resulted in the first profound and durable responses for patients with metastatic disease.[1,2] Immunotherapy is now standard of care for many solid organ cancers including lung, hepatocellular, and gastric cancers.[3] However, for some primary organ sites, such as pancreatic ductal adenocarcinoma, epithelial ovarian, and biliary tract cancers, the results of immune checkpoint inhibitors (ICIs) have been disappointing.[4–7] The reasons for this are multifactorial and include a hostile tumor microenvironment (TME) comprising desmoplastic stroma, poor immune cell penetrance, alternative immune checkpoints, and immunosuppressive myeloid cells.[8] Relieving immunosuppression of the TME has been postulated as a promising strategy to improve the clinical efficacy of cancer therapeutics,[9] with myeloid-derived suppressor cells (MDSCs) representing a crucial immunosuppressive barrier to overcome in order to increase the chances of biological and clinical responses to treatment.[10]

MTL-CCAAT enhancer-binding protein alpha (CEBPA) is a first-in-class small activating RNA (saRNA) therapy targeting the upregulation of the transcription factor C/EBP-α, a master regulator of the myeloid cell lineage.[11] Decreased C/EBP-α expression frequently occurs in the context of cancer, and loss of C/EBP-α causes a block in myeloid differentiation, leading to an accumulation of immunosuppressive MDSCs.[12–17] The saRNA is packaged within myeloid-targeting liposomes and, when delivered to myeloid cells, is proposed to restore C/EBP-α protein to normal levels to drive differentiation of immature myeloid cells such as MDSCs, rendering the TME less immunosuppressive. MDSCs are associated with resistance to many therapy classes, including tyrosine kinase inhibitors. In OUTREACH, a first-in-human (FIH) phase 1 clinical trial of MTL-CEBPA in combination with sorafenib among patients with hepatocellular carcinoma (HCC), it was found that the combination treatment was associated with both durable and complete tumor responses, and there was evidence of reduced myeloid-driven immunosuppression in the TME and periphery suggesting that MTL-CEBPA may increase the effectiveness of sorafenib therapy in HCC.[18–20]

Indeed, MDSCs are associated with both primary and secondary immune checkpoint blockade resistance[21] and represent an active target for improving ICI clinical efficacy.[9,22] In pre-clinical studies, combination treatment with MTL-CEBPA and an anti-PD-1 antibody showed marked synergistic abrogation of tumor progression.[23] Further, we found that MTL-CEBPA abrogates the immune suppressive activity of TAMs and MDSCs in this syngeneic mouse model enabling CD8 T cell-driven anti-tumor activity, indicating that MTL-CEBPA removes the immunosuppressive breaks required for ICI action on CD8 T cells.[20] Thus, in the TIMEPOINT clinical trial, we evaluated the effect of MTL-CEBPA in combination with an anti-PD-1 antibody, pembrolizumab, in patients with advanced solid organ malignancies known to present with ICI resistance and performed mechanistic studies to determine the associated changes in the TME in addition to patient characteristics that may be associated with clinical outcome to this combination therapy.

RESULTS

Overall approach and cohort characteristics

TIMEPOINT is a phase 1a+b, dose-escalation and expansion study between November 2019 and March 2022 aimed to primarily determine the safety, tolerability, and efficacy of MTL-CEBPA in combination with the anti-PD-1 antibody, pembrolizumab (Table 1). Secondary to this, an exploratory multimodal approach was used to characterize the biological and pharmacodynamic effects of MTL-CEBPA in combination with pembrolizumab on the TME, as well as to understand whether patients with certain characteristics are associated with patient outcomes. Patients recruited to TIMEPOINT represent the typical phase 1 range of cancer types with advanced stage and heavily pre-treated disease in both phase 1a and phase 1b parts of the study. The dosing regime is described in Figure 1A, and the baseline demographics of the 50 patients who received at least one dose of study treatment and were evaluable for safety analysis at the study cutoff are presented in Tables S1 and S2.

MTL-CEBPA in combination with pembrolizumab is safe and tolerable at all dose levels studied

Evaluation of safety across all patients in the dose-finding 1a part of the study demonstrated no dose-limiting toxicity, serious treatment-emergent adverse events (TEAEs), or Common Terminology Criteria for Adverse Events (CTCAE) grade $\geq$3 TEAE related to MTL-CEBPA only regardless of dose. The MTL-CEBPA dose of 130 mg/m^2 weekly for 3 out of 4 weeks was confirmed as the recommended dose and schedule for the expansion 1b part of the study. A total of 7 unique patients reported serious adverse events (SAEs) in phase 1b, comprising atrial fibrillation, upper abdominal pain, large intestinal obstruction, vomiting, COVID-19, muscular weakness, allergic encephalitis, and female genital tract fistula. Only the event of allergic encephalitis was considered related to the study intervention, with the event considered possibly related to both MTL-CEBPA and pembrolizumab treatment. In phase 1a, 2 TEAEs (adrenal insufficiency and hepatocellular injury) led to the discontinuation of pembrolizumab treatment, while in phase 1b, another patient had 2 TEAEs that led to the discontinuation of pembrolizumab (alanine transaminase increased and aspartate transaminase increased). In phase 1b only, 2 patients had TEAEs that led to the discontinuation of both MTL-CEBPA and pembrolizumab (fatigue and allergic encephalitis); however, there were no TEAEs leading to the discontinuation of MTL-CEBPA only treatment. Dose interruptions were infrequent, and no patients in either phase had TEAEs that led to a dose reduction of MTL-CEBPA, pembrolizumab, or dose reduction of both study interventions.

MTL-CEBPA in combination with pembrolizumab demonstrates clinical activity in intrahepatic cholangiocarcinoma

Among 40 patients evaluable for Response Evaluation Criteria in Solid Tumors (RECIST) criteria, four patients in the study had a confirmed partial response (PR, response rate: 10%). Disease control rates of 80.0% and 57.1%, respectively, were reported for patients with intrahepatic cholangiocarcinoma and ovarian

CellPress
OPEN ACCESS

Table 1. Most common adverse events (in at least 10% of patients) and CTCAE grade ≥ 3

	All patients Ph1a *N* = 10		All patients Ph1b *N* = 40		All patients Ph1a and Ph1b *N* = 50	
MedDRA preferred term	TEAE	≥3 CTCAE	TEAE	≥ 3 CTCAE	TEAE	≥ 3 CTCAE
Patients with TEAE/CTCAE grade ≥ 3 TEAE	10 (100.0)	3 (30.0)	39 (97.5)	17 (42.5)	49 (98.0)	20 (40.0)
Anemia	4 (40.0)	0	13 (32.5)	1 (2.5)	17 (34.0)	1 (2.0)
Fatigue	5 (50.0)	0	12 (30.0)	1 (2.5)	17 (34.0)	1 (2.0)
Abdominal pain	1 (10.0)	0	13 (32.5)	2 (5.0)	14 (28.0)	2 (4.0)
Alanine aminotransferase increased	4 (40.0)	2 (20.0)	8 (20.0)	0	12 (24.0)	1 (2.0)
Aspartate aminotransferase increased	3 (30.0)	1 (10.0)	9 (22.5)	1 (2.5)	12 (24.0)	2 (4.0)
Decreased appetite	3 (30.0)	0	8 (20.0)	0	11 (22.0)	0
Nausea	5 (50.0)	0	6 (15.0)	0	11 (22.0)	0
Back pain	2 (20.0)	0	7 (17.5)	0	9 (18.0)	0
Constipation	2 (20.0)	0	7 (17.5)	0	9 (18.0)	0
Diarrhea	4 (40.0)	0	5 (12.5)	0	9 (18.0)	0
Gamma-glutamyltransferase increased	2 (20.0)	0	6 (15.0)	2 (5.0)	8 (16.0)	2 (4.0)
Vomiting	1 (10.0)	0	7 (17.5)	1 (2.5)	8 (16.0)	1 (2.0)
Blood alkaline phosphatase increased	3 (30.0)	0	4 (10.0)	1 (2.5)	7 (14.0)	1 (2.0)
Arthralgia	3 (30.0)	0	3 (7.5)	0	6 (12.0)	0
Cough	2 (20.0)	0	4 (10.0)	0	6 (12.0)	0
Lethargy	0	0	6 (15.0)	0	6 (12.0)	0
Abdominal pain upper	2 (20.0)	0	3 (7.5)	1 (2.5)	5 (10.0)	1 (2.0)
Hyponatremia	1 (10.0)	0	4 (10.0)	1 (2.5)	5 (10.0)	1 (2.0)
Lower respiratory tract infection	3 (30.0)	0	2 (5.0)	0	5 (10.0)	0

cancer. The best objective response according to the treatment group is summarized in Table S3, Figures 1B and S1A. Patients with confirmed partial responses had epithelioid mesothelioma, high-grade serous ovarian cancer, advanced metastatic atypical lung neuroendocrine tumor with multiple metastatic disease, and advanced intrahepatic cholangiocarcinoma and were heavily pre-treated with at least 3 lines of systemic therapy.

Confirmed partial response was seen in a patient with epithelioid mesothelioma that had prior carboplatin, cisplatin, and pemetrexed chemotherapy. The patient achieved PR in cycle 4 with 83% target lesion reduction with a duration of response of approximately 17 months, progression-free survival (PFS) of 21 months, and ongoing study treatment at data cutoff (DCO) after oligometastasis surgery. The second case was a patient with high-grade serous ovarian cancer with six previous lines of systemic therapy including bevazicumab, carboplatin, doxorubicin, gemcitabine, olaparib, and paclitaxel. The patient had a PR with a maximum of 69% tumor size reduction before disease progression at the end of cycle 6. The third case was a patient with an advanced metastatic atypical lung neuroendocrine tumor with multiple metastatic disease and three prior lines of systemic anti-cancer systemic treatment. The patient achieved a PR in cycle 4 and is ongoing study treatment at DCO (after oligometastasis surgery). The fourth case was a patient with advanced intrahepatic cholangiocarcinoma who achieved a PR in cycle 2 and was in remission at DCO; however, the patient discontinued both study drugs due to a Common Terminology Criteria grade 4 SAE of immune-related encephalitis considered possibly related to study treatments.

Pharmacokinetics of MTL-CEBPA with pembrolizumab

Over the MTL-CEBPA dose range, there was approximately a 2.2-fold and 1.9-fold increase in Cmax and AUCinf, respectively, after the first dose of MTL-CEBPA. Dose-proportional pharmacokinetics is also observed after the second dose of MTL-CEBPA, with a 2.6-fold and 1.9-fold increase in Cmax and AUC 0–72 h, respectively, over this dose range (Figure 1C). Pembrolizumab will still be present in plasma when the second dose of MTL-CEBPA is administered; after the second dose of MTL-CEBPA, there was a 24%–38% reduction in Cmax and a 12%–52% reduction in AUC 0–72 h when compared with the first dose. However, this is consistent with data obtained after MTL-CEBPA monotherapy over this dose range. Although no formal statistical comparison has been performed, based on the data from this study, pembrolizumab does not appear to affect the pharmacokinetics of CEBPA-51.

Patients treated with MTL-CEBPA demonstrate target pharmacodynamic response

Upregulation of CEBPA gene expression is the proposed mechanism of action of MTL-CEBPA, and we observed CEBPA RNA upregulation in peripheral white blood cells from patients treated with MTL-CEBPA where a biospecimen was available ($n = 39$, Figure 2A). 62% patients had a minimum 10% increase of CEBPA RNA 24 h after MTL-CEBPA administration. This increase in CEBPA RNA expression can be attributed to MTL-CEBPA (as pembrolizumab was administered following when this sample was taken), either due to direct increases in RNA levels or changes in peripheral white blood cell subsets. A significant upregulation of

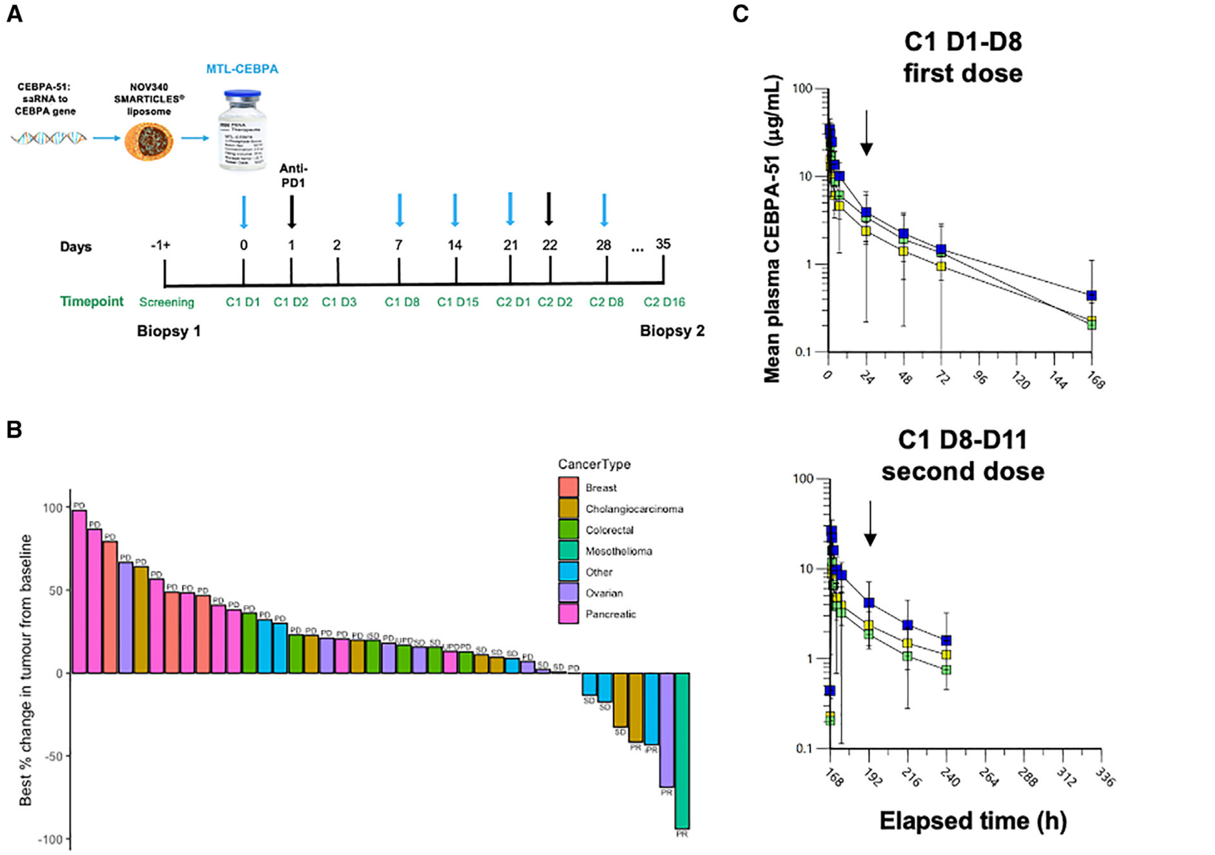

Figure 1. Best objective tumor response and pharmacokinetics of MTL-CEBPA in combination with pembrolizumab
(A) Dosing schedule of MTL-CEBPA/pembrolizumab in the TIMEPOINT clinical trial.
(B) Waterfall plot illustrating patient best objective tumor response across treatment groups in phase 1a and phase 1b. Note: an i prefix represents a response evaluated according to the iRECIST or irRECIST criteria. Patients with no post-baseline RECIST assessments are excluded from this figure. iRECIST, immune RECIST; irRECIST, immune-related RECIST; PD, progressive disease; PR, partial response; SD, stable disease; UPD, unconfirmed progressive disease.
(C) Plasma CEBPA-51 concentration versus time profiles were collected over 7 days after the first dose of MTL-CEBPA (left) and over 3 days after the second dose of MTL-CEBPA (right). Cohort: 70 mg/m^2 N = 4, 98 mg/m^2 N = 3, 130 mg/m^2 N = 9. Black arrow denotes pembrolizumab dose.

CEBPA protein was detected in enriched monocytes from peripheral blood after MTL-CEBPA infusion in patients (Figure S2A), but not in the enriched lymphocyte fraction, in line with the known preferential delivery of NOV340 to myeloid cells.

MTL-CEBPA in combination with pembrolizumab drives increased immune activity in the TME and reduces immunosuppressive myeloid populations

To assess the biological and pharmacodynamic effects of MTL-CEBPA in combination with pembrolizumab on the TME, we performed a multimodal exploratory analysis of 24 patients treated with MTL-CEBPA and pembrolizumab, for whom a pre- and post-tumor biopsy was available. The assays performed on each patient and their tumor type are captured in Table S4. Where the patient was not evaluable for RECIST, best objective response was used for clinical activity assessment.

Transcriptional analyses of pre- and post-treatment biopsies using the Nanostring PanCancer IO 360 platform indicated significant pathway enrichment of genes associated with enhanced T cell priming and activation, T stem cell memory and cytotoxicity, as well as increased cancer antigen recognition and upregulation of genes associated with myeloid cell activity (Figures 2B, S2B, and S2C; Table S5). Transcriptional increases in immune cell activation were validated at the cellular level in 23 patients with paired biopsies, using multiple Brightplex immunohistochemistry (IHC) panels, as patients had significant increases in total CD8 T cells and proliferating cytotoxic T cells in the TME post treatment (Figure 2C).

Also concurrent with transcriptional evidence of reduced immunosuppression and as seen previously in the clinical trial, OUTREACH, there was a significant decrease in the proportion of M2 macrophages after treatment (Figure 2D) and also a non-significant reduction in the proportion and numbers of intratumoral PMN-MDSCs post treatment with MTL-CEBPA and anti-PD-1 in patients with >1 PMN-MDSCs/mm^2 at baseline (Figure 2E). Furthermore, as observed previously in OUTREACH, there was a trend reduction in peripheral blood PMN-MDSCs post treatment (Figures 2F and S2D).[20,24,25] Collectively, these data suggest that the combination of MTL-CEBPA and pembrolizumab can promote increased T cell infiltration, activity,

CellPress
OPEN ACCESS

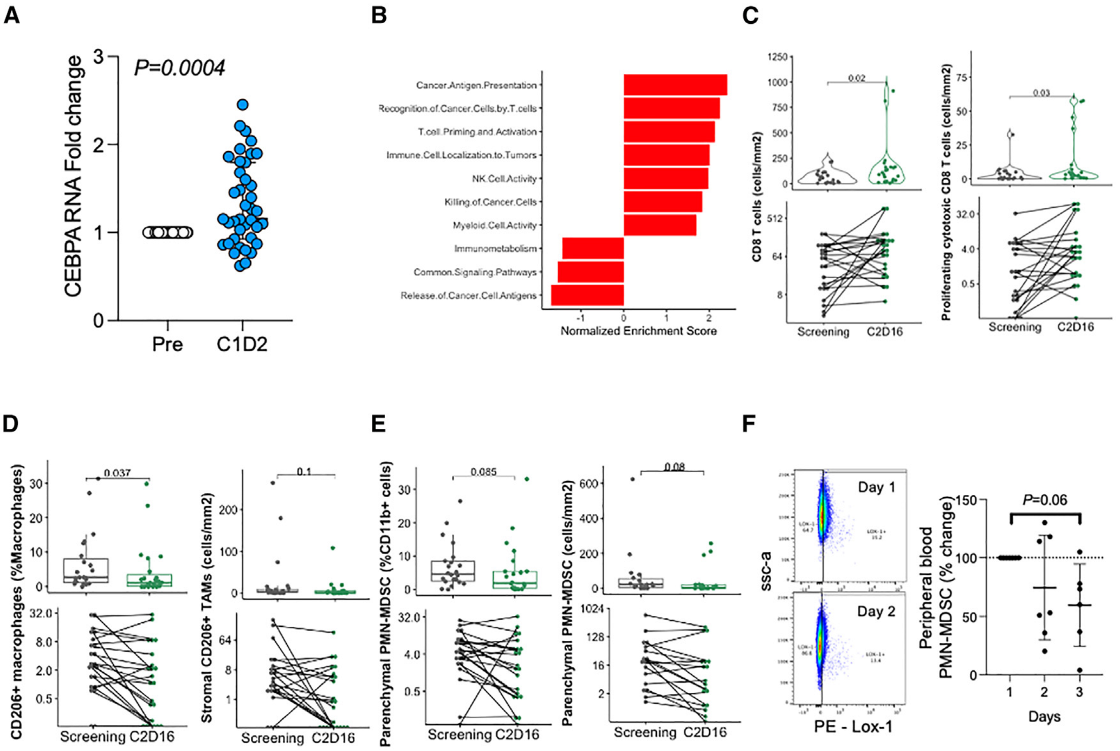

Figure 2. MTL-CEBPA and pembrolizumab combination treatment is associated with changes across tumor types, consistent with immune inflammation and activation

(A) Fold change of CEBPA RNA at C1 D2 normalized to pre-treatment values (C1 D1). Bar is at median with 95% confidence error bars, each point is one patient. Significance was determined by one sample Wilcoxon test with hypothetical median value of 1.

(B) Fast gene set enrichment analysis (FGSEA) analysis of differentially expressed genes between pre- and post-treatment time points across all patients, testing Nanostring pathways of the IO Pan-cancer panel. Pathways in red reached significance (*p* adjust <0.05), with significance determined and adjusted for multiple testing by the fgsea algorithm.

(C) Quantification of CD3^{+} CD8^{+} T cell numbers (left) and CD3^{+}CD8+Ki67+GZMB+ T cell numbers (right) between pre- (screening) and post treatment (C2D16) of 23 paired biopsies as determined by IHC. *p* values are non-adjusted and are determined by paired two-sided Wilcoxon test.

(D) Quantification of CD11b+CD68^{+}CD64^{-}CD206+CD163^{-} cells as a proportion of total macrophages (CD11b+CD68^{+} cells) (left) and stromal CD11b+CD68^{+}CD64^{-}CD206+CD163^{-} cell numbers (right) between pre- (screening) and post-treatment (C2D16) of 23 paired biopsies as determined by IHC. *p* values are non-adjusted and are determined by paired two-sided Wilcoxon test.

(E) Quantification of parenchymal CD11b+CD15^{+}CD14^{-}HLA-DR^{-}LOX1+ (PMN-MDSC) cells as a proportion of total myeloid cells (CD11b+ cells) (left), between pre- (screening) and post treatment (C2D16) of 23 paired biopsies as determined by IHC. Right, parenchymal PMN-MDSC cell numbers in patients with at least 1 PMN-MDSC per cell/mm^2 ($N = 18$). *p* values are non-adjusted and are determined by paired two-sided Wilcoxon test. The boxplots in (C), (D), and (E) show the data distribution, where the line denotes the median, the box edges show the interquartile range, and each dot is one patient.

(F) Flow cytometric detection of PMN-MDSCs in the blood of TIMEPOINT patients. % change refers to change of PMN-MDSC as a proportion of total live cells (see gating strategy in Figure S2D) compared to C1 D1. Each point is one patient, line is at mean with SD error bars. Significance was determined by Wilcoxon test compared to expected value of 100.

and a reduction in myeloid cells with an immunosuppressive phenotype in the TME and periphery, key steps for promoting anti-tumor immunity.[26]

Increased T cell infiltration and conversion of an immune-desert to immune-inflamed TME is associated with MTL-CEBPA and pembrolizumab treatment

There was significant heterogeneity in the baseline distribution of CD8 T cells pre-treatment between patient biopsies, and tumors that had lower TILs at baseline demonstrated the greatest increase in both total CD8 T cells and cytotoxic CD8 T cells post treatment when split based on median value (Figure 3A), suggesting the possibility of two groups of patients with distinct pre-treatment biopsy immune phenotypes and biological responses to treatment. To explore this notion, we split the 23 patients by the midpoint of a pre-defined transcriptional gene signature, Immunosign-21 (IS21), that captures T cell infiltration, activation, and cytotoxicity.[27,28] High IS21 scores at baseline indicate those tumors that were immune-infiltrated or immune-inflamed (hot) pre-treatment, where those with IS21 < 10 are likely to have been immune-excluded or immune-desert (cold). In line with this, at baseline, hot tumors contained significantly greater CD8 T cell numbers and higher PDL1 expression than cold tumors (Figures S3A and S3B).

Patients with cold tumors at baseline demonstrated significant increases in IS21 gene expression signature post

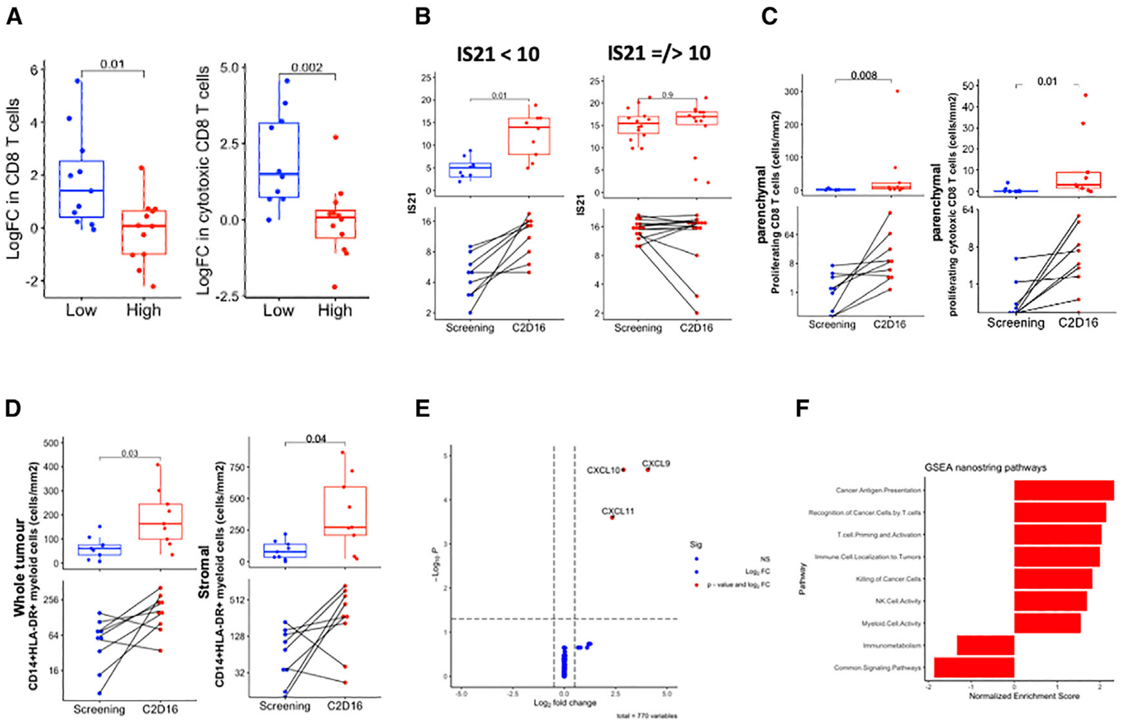

Figure 3. MTL-CEBPA/pembrolizumab combination treatment is associated with immunomodulatory changes in the immune-desert TME, converting to immune-inflamed

(A) Fold change in $CD3^+CD8^+$ T cells (left) and $CD3^+CD8^+$GZMB+ T cells (right) at C2D16 compared to screening, between patients with low or high levels of TIL at baseline using the median as threshold at screening. *p* value was determined by an unpaired two-sided Wilcoxon test.

(B) Quantification of Immunosign, IS21 between pre- (screening) and post treatment (C2D16) between cold and hot tumors, left and right, respectively. *p* values are non-adjusted and were determined by paired two-sided Wilcoxon test.

(C) IHC quantification of parenchymal $CD3^+CD8^+Ki67$+ T cells (left) and $CD3^+CD8^+$Ki67+GZMB+ T cells (right) between pre- (screening) and post treatment (C2D16) of 9 paired biopsies with an immune-desert TME (IS21 < 10). *p* value is determined by paired two-sided Wilcoxon test.

(D) IHC quantification of CD11b+$CD14^+CD15^-$HLA-DR+ cells in the whole tumor (left) or stroma (right) between pre- (screening) and post treatment (C2D16) of 9 paired biopsies with an immune-desert TME (IS21 < 10). *p* value is determined by paired two-sided Wilcoxon test. The boxplots in (A)–(D) show the data distribution, where the line denotes the median, the box edges show the interquartile range, and each dot is one patient.

(E) Differential gene expression calculated between pre- and post-treatment time points of 9 paired immune-desert patient tumor biopsies. *p* values on y axis refer to adjusted *p* values by DESeq2 algorithm.

(F) FGSEA analysis of differentially expressed gene sets between pre- (screening) and post treatment (C2D16) of 9 paired biopsies with an immune-desert TME (IS21 < 10), using supplied Nanostring pathways of the IO Pan-cancer panel. Pathways in red reached significance (*p* adjust <0.05), adjusted by fgsea algorithm.

treatment, unlike patients with hot tumors at baseline (Figure 3B). In fact, 6 out of the 9 immune-desert tumors were reclassified as immune-inflamed post treatment. These transcriptional changes in immune-desert tumors were accompanied by significant increases in parenchymal T cell subsets, including proliferating CD8 T cells, proliferating cytotoxic CD8 T cells, proliferating PD1+ CD8 T cells, and non-CD8 cytotoxic T cells (Figures 3C and S3C). Concurrent with enhanced T cell immunity, in immune-cold tumors, we observed a significant increase in CD11b+$CD14^+CD15^-$HLA-DR+ antigen-presenting cell (APC)-like myeloid cells at C2D16 and upregulation of T cell chemokines, CXCL9, CXCL10, and CXCL11 RNA, in the TME compared with the screening biopsies (Figures 3D and 3E), suggesting an increase in both antigen presentation and inflammatory activity as supported by gene set enrichment of these pathways alongside T cell memory gene signatures (Figures 3F and S3D). The increase in stromal HLA-DR+ APC-like myeloid cells demonstrated a non-significant correlation with the increase in proliferating cytotoxic T cells post treatment in the TME (Figure S3E). Collectively, these data suggest that MTL-CEBPA in combination with an anti-PD-1 antibody may promote increased immune infiltration and activity within previously immune-cold tumors.

Similar patterns of adaptive immune activity associated with treatment were also detected in the group of 14 patients with immune-inflamed tumors; the enriched gene pathways were similar to those seen in cold tumors, including increased natural killer, T cell, and myeloid cell activity (Figures 4A and 4B). However, CD8 T cell infiltration was less than in cold tumors (Figure 3A), and only non-significant increases in stromal APC-like myeloid cell infiltration were detected, while T cell chemokines did not significantly change (Figures S4C and S4D). Of interest, the rate of disease stabilization between the two groups was slightly higher in patients with immune-desert tumors at baseline, compared to those with immune-inflamed tumors (immune-desert, 33%; immune-inflamed, 21.4%).

CellPress
OPEN ACCESS

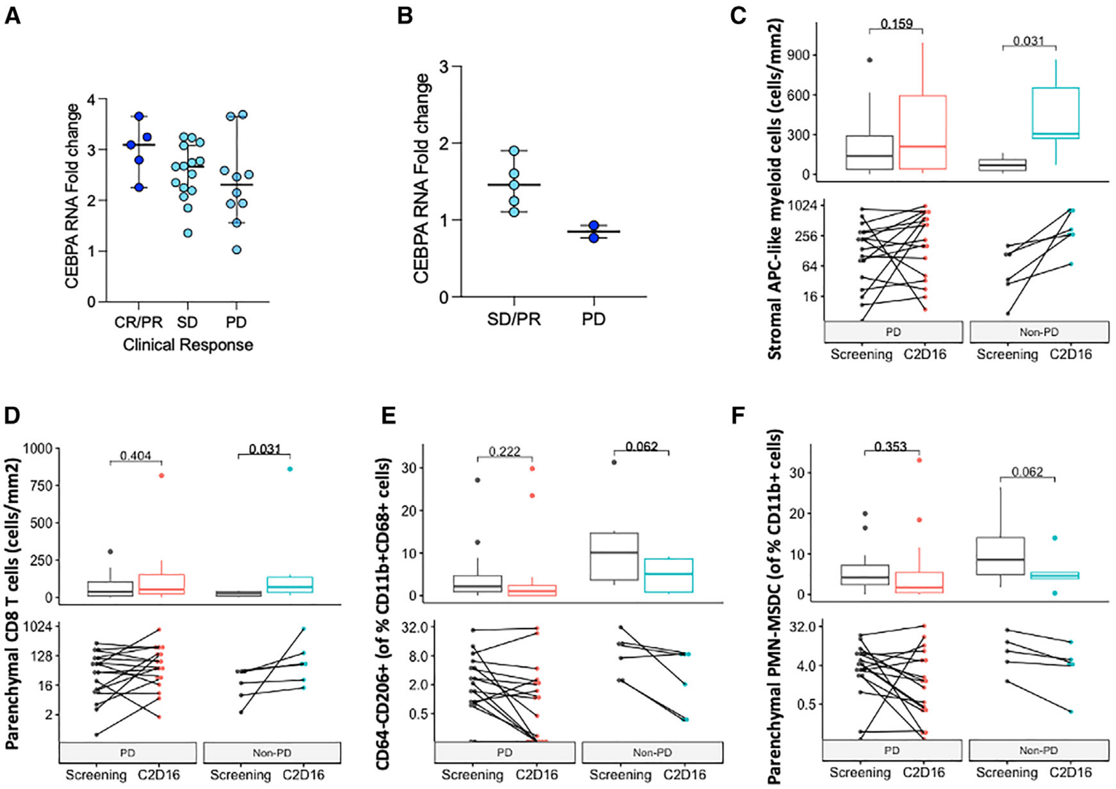

Figure 4. Characteristics linking to MTL-CEBPA mode of action are enriched in patients with stable disease after combination treatment

(A) Fold change of CEBPA RNA at C1 D2 compared to pre-treatment, C1 D1 in patients with HCC from the OUTREACH clinical trial. Bar is at median with 95% confidence interval error bars, each point is one patient. Significance was tested with Kruskal-Wallis test with *p* value determined 0.1375.

(B) Fold change of CEBPA RNA at C1 D2 normalized to pre-treatment, C1 D1 in iCCA patients from TIMEPOINT. Bar is at median with 95% confidence interval error bars, each point is one patient. Significance was not tested due to low patient numbers. For (A) and (B), clinical outcome refers to clinical response by RECIST or if unavailable, at C2 D22 determined by site.

(C) Change in stromal CD11b+CD14$^+$CD15$^-$HLA-DR+ APC-like cells between pre- (screening) and post treatment (C2D16) calculated independently for patients with disease stabilization and progressive disease. Significance was determined by paired two-sided Wilcoxon test and *p* values are unadjusted.

(D) Change in numbers of parenchymal CD8 T cells (CD3$^+$CD8$^+$) between pre- (screening) and post treatment (C2D16) in patients with disease stabilization and progressive disease independently calculated. Significance was determined by paired two-sided Wilcoxon test and *p* values are unadjusted.

(E) Change in proportion of CD11b+CD68$^+$CD64$^-$CD206+CD163$^-$ cells as a proportion of total macrophages (CD11b+CD68$^+$ cells) between pre- (screening) and post treatment (C2D16) in patients with disease stabilization and progressive disease independently calculated. Significance was determined by paired two-sided Wilcoxon test and *p* values are unadjusted.

(F) Change in proportion of parenchymal CD11b+CD14$^-$CD15+HLA-DR-LOX1+ cells (PMN-MDSCs) as a percentage of total myeloid cells (CD11b+) between pre- (screening) and post treatment (C2D16) in patients with disease stabilization and progressive disease calculated independently. Significance was determined by paired two-sided Wilcoxon test and *p* values are unadjusted. The boxplots in (C)–(F) show the data distribution, where the line denotes the median, the box edges show the interquartile range, and each dot is one patient (*N* = 23). Clinical outcome of PD or non-PD refers to the clinical response determined by site at C2 D22.

In-depth understanding of the biological characteristics that are related to treatment response helps identify the patients most likely to benefit from therapy. The proposed mode of action of MTL-CEBPA is to induce CEBPA upregulation in immature immunosuppressive myeloid cells, leading to myeloid cell differentiation, relieving immunosuppressive myeloid activity in the TME.[20] Retrospective analysis of RNA from the OUTREACH clinical trial of patients with HCC demonstrated a non-significant trend between increased CEBPA expression post MTL-CEBPA and clinical outcome (Figure 4A). Although this observation was not validated across the pan-cancer cohort (Figure 5A), in the small cohort of TIMEPOINT patients with intrahepatic cholangiocarcinoma, patients who demonstrated CEBPA upregulation 24 h after MTL-CEBPA treatment demonstrated disease stabilization post combination treatment compared to those with no upregulation of CEBPA (Figure 4B).

Other treatment-related biological changes were associated with clinical outcomes; the increase in APC-like inflammatory myeloid cells and CD8 T cells post treatment were significant in patients that showed disease stabilization but not in those with progressive disease (PD) (Figures 4C and 4D). Similarly, the trend reduction in proportions of immunosuppressive myeloid cells was only true in patients with disease stabilization post treatment and lost in those with PD (Figures 4E and 4F).

Thus, we investigated whether a greater presence of these MTL-CEBPA target cells before treatment is associated with clinical outcome.

Patients with a greater presence of myeloid-associated immunosuppression are more likely to have disease stabilization post MTL-CEBPA combination treatment

Patients with greater proportions of M2-like TAMs at baseline were significantly more likely to demonstrate disease stabilization post treatment, with a similar non-significant trend seen for PMN-MDSCs (Figures 5B and 5C). Furthermore, differential gene expression analysis of baseline pre-treatment tumor biopsies between patients with PD and non-PD identified a 15 gene signature associated with clinical outcome, named here as MTL-CEBPA, Checkpoint Gene Signature or CCGS (Figures 5A and 5B). Several of the genes relate to myeloid-driven immunosuppression, hinting to the mechanism of action of MTL-CEBPA, including histidine decarboxylase, arginase 2, interleukin-1 beta, and WNT5. CCGS correlated with the percent change in tumor size after treatment, and patients with higher CCGS scores had greater PFS (Figures 5C, 5D, and S5D).

As TIMEPOINT is a combination treatment study of MTL-CEBPA with pembrolizumab, we considered whether the CCGS was associated with response to the anti-PD-1 antibody treatment alone. However, there was no relationship between CCGS score and clinical outcomes after treatment in a pembrolizumab-only dataset, indicating that the association of CCGS score with disease stabilization after treatment was specific to the combination treatment of MTL-CEBPA and pembrolizumab, rather than pembrolizumab alone (Figure S5F). Furthermore, traditional and commonly used biomarkers of pembrolizumab response such as PDL1 expression were not associated with disease stabilization after the combination treatment (Figures 5A and S5E).[29–32]

Finally, liquid biopsy-based biomarkers are attractive given their non-invasive nature. We therefore tested whether we could detect a circulating proteomic signature associated with clinical outcome after MTL-CEBPA/pembrolizumab combination using baseline plasma samples. Using the OLINK platform, several proteins were associated with disease stabilization, including higher levels of plasma DNER and lower levels of 4EBP1, IL15RA, tumor necrosis factor, IL2RB, and CSF1 (Figures 5E, 5F, and S5G), with a combined signature of these proteins demonstrating that patients with a lower score were more likely to not progress after treatment with the combination (Figure 5E). We performed a similar plasma protein analysis on baseline pre-treatment samples from the OUTREACH clinical trial, and, interestingly, we observed non-significant trends for 3 of these proteins (Figure S5H). Collectively, these data suggest that certain patterns of circulating proteins may be associated with clinical outcomes after MTL-CEBPA combination treatments, across multiple tumor types and combination drugs.

Exploratory analysis identified multiple biological characteristics that were associated with clinical outcome after combination treatment with MTL-CEBPA and pembrolizumab (Figures 5 and S5). Univariable Cox regression analysis of these characteristics with PFS confirmed that the plasma protein signature and CCGS were associated with disease stabilization (Table 2). The type of cancer also demonstrated a significant association with disease progression for breast cancer and pancreatic cancer specifically, while other demographic characteristics did not. Multivariable regression indicated that CCGS and plasma protein signature were not independent of tumor type, suggesting that these signatures may perform in certain cancer types.

DISCUSSION

This study evaluated the safety of a saRNA therapy in combination with checkpoint blockade. MTL-CEBPA, the first saRNA therapy to enter clinical trials, is proposed to produce an immune-modulatory effect via upregulation of the transcription factor *CEBPA*, leading to the modulation of the myeloid cell lineage and differentiation, subsequently affecting myeloid cells in the periphery and TME.[20] TIMEPOINT is a phase 1 clinical study including patients with a very wide range of advanced, heavily pre-treated cancers largely expected to be resistant to any treatment, including single-agent ICIs. Consistent with previous clinical data of the FIH OUTREACH trial, MTL-CEBPA has an overall favorable and manageable safety profile when used as a combination agent, in this case with pembrolizumab. The overall clinical activity observed in the study was largely in line with the advanced resistant stage and heavily pre-treated cancer. We also observed interesting radiological and biological responses and disease stabilization in patients with advanced, difficult to treat cancer, which may provide insight and direction for subsequent proof-of-concept studies.

As observed in real-world tumors, patients present with cancers that reflect various levels of immune cell composition and infiltration. These are broadly classified into immune-desert (cold), immune-excluded, and immune-infiltrated, or inflamed (hot) tumors. Generally, immune-inflamed tumors, i.e., those with pre-existing CD8 T cell infiltration, are more likely to respond to checkpoint inhibitors than immune-cold tumors.[33–36] Thus, efforts to try to stimulate the egress of T cells into the TME as an adjuvant to checkpoint inhibitors is a shared goal of the oncology field.[37,38] Collectively, our data use polyorthogonal approaches to demonstrate that the combination of MTL-CEBPA with pembrolizumab is associated with an increase in T cells across tumor types, though this response was greater in patients with an immune-cold TME at baseline. Indeed, in these tumors, we observed increased immune activity leading to changes of the TME toward an immune inflamed in the majority of these tumors. Without inclusion of a monotherapy control group with anti-PD-1 only, it is impossible to discount the possibility that pembrolizumab alone could have driven these changes; thus, further investigation is required to understand whether these changes can be driven by upregulation of CEBPA expression in immature myeloid cells, directing cell differentiation toward an inflammatory, antigen-presenting phenotype.

In an analysis of patients with hepatocellular carcinoma treated with MTL-CEBPA and sorafenib, we observed a trend of CEBPA mRNA upregulation associated with outcome, though we did not detect an association between CEBPA mRNA upregulation with outcome in TIMEPOINT. There was an interesting trend in intrahepatic cholangiocarcinoma tumors whereby patients with upregulation of CEBPA RNA 24 h after drug administration appeared more likely to have a response or disease

CellPress
OPEN ACCESS

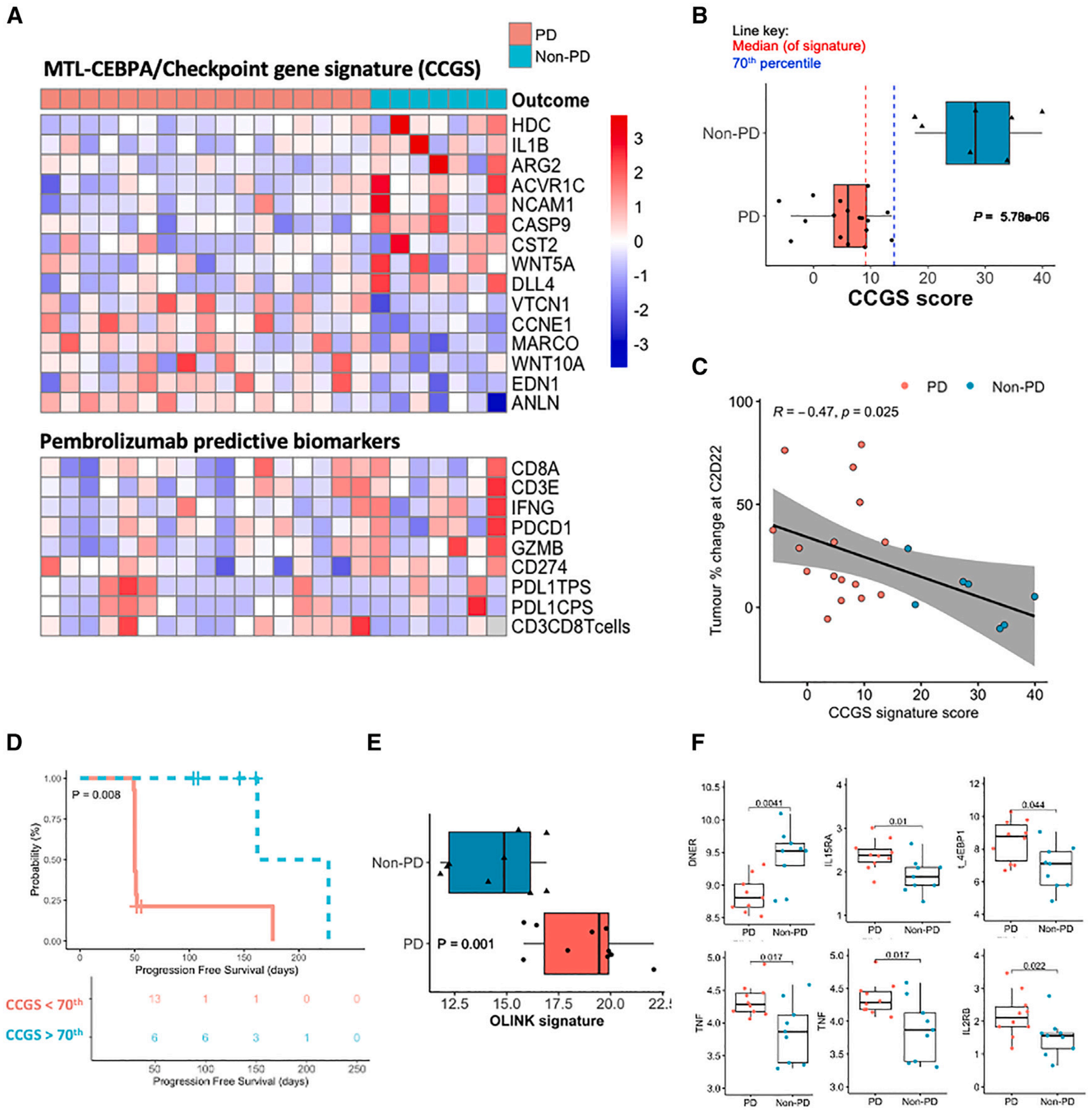

Figure 5. Predictive transcriptomic- and proteomic-based signatures of clinical outcome

(A) VST-RUVg visualization of CCGS gene expression (top) and pembrolizumab-related biomarkers (bottom) pre-treatment in 24 patient biopsies, split by progressive disease (red; PD) and non-progressive disease (blue; non-PD).

(B) CCGS quantification at pre-treatment between patients with PD and non-PD post treatment. Significance was determined with two-sided Wilcoxon test. Each dot represents one patient ($N = 24$).

(C) Spearman correlation of CCGS at pre-treatment in patient tumor biopsies with % tumor change from baseline at C2 D22 after two cycles of treatment, each point is one patient.

(D) Kaplan-Meier plot of progression-free survival with CCGS score, splitting patients by 70th percentile of CCGS. Data are censored as indicated by crosses. Significance was determined by log-rank test.

(E) Combined signature of OLINK analysis at pre-treatment between patients with PD and non-PD by site at C2 D22. Significance was determined with an unpaired two-tailed Wilcoxon test. Each dot represents one patient.

(F) OLINK plasma analysis of circulating proteins using Inflammation panel of patient plasma at baseline (pre-treatment) in TIMEPOINT (top) as NPX values. Clinical outcome of PD or non-PD refers to the clinical response determined by site at C2 D22 for (A)–(F). Significance was determined with an unpaired two-tailed Wilcoxon.

p values are non-adjusted. The boxplots in (B), (E), and (F) show the data distribution, where the center line denotes the median, the box edges show the interquartile range, and each point is one patient.

Table 2. Regression analyses of TIMEPOINT variables

	Univariable Cox regression			Multivariable Cox regression		
	HR (95% CI for HR)	*N* included in analyses	*p*	HR (95% CI for HR)	*N* included in analysis	*p*
CEBPA mRNA	1.2 (0.61–2.3)	30	0.61	–	–	NA
CCGS	0.93 (0.88–0.99)	20	0.021	0.88 (0.76–1)	13	0.45
OLINK signature	1.8 (1.2–2.6)	16	0.003	1 (0.72–1.4)	13	0.08
CD206+CD163-M2%	0.94 (0.86–1)	20	0.22	–	–	NA
PMN-MDSC	0.94 (0.85–1)	20	0.24	–	–	NA
Phase 1a vs. 1b	1.4 (0.57–3.4)	40	0.48	–	–	NA
Gender	1.4 (0.64–3)	40	0.41	–	–	NA
Cancer type (vs. other)	–	40	–	–	13	
Breast	11.7 (2.22–61.65)		0.004	NA		1
CCA	2.1 (0.47–9.30)		0.33	0.0000004 (0–Inf)		1
Colon	2.3 (0.24–21.71)		0.48	0.00000001 (0–Inf)		1
Ovarian	2.0 (0.57–6.80)		0.28	0.000002 (0–Inf)		1
Pancreatic	4.1 (1.06–16.44)		0.04	0.0000002 (0–Inf)		1
Rectal	4.5 (0.89–22.97)		0.07	0.0000004 (0–Inf)		1
Dose (vs. low)	–	40	–	–	–	NA
Mid	3.9 (0.61–25.31)		0.15	–	–	–
High	2.2 (0.50–9.77)		0.3	–	–	–
Ethnicity (vs. white)	–	39	–	–	–	NA
Asian/Asian British	0.7 (0.25–2.17)		0.58	–	–	–
Black/Black British	2.8 (0.65–12.27)		0.17	–	–	–
Other	3.5 (0–Inf)		1	–	–	–

stabilization, and we saw higher disease control rates in this subgroup (disease control rate by RECIST = 80%). Previous studies of biliary tract cancers with single-agent pembrolizumab (KEYNOTE 158 and KEYNOTE 028) demonstrated disease control rates of approximately 25%.[4] Additional data with greater patient numbers per group are required to understand how the relationship between the on-target mechanism of action of MTL-CEBPA may be linked to clinical outcome in certain tumor types. Due to the lack of association of CEBPA upregulation with clinical outcome in TIMEPOINT, we cannot conclude that MTL-CEBPA is solely responsible for the biological responses or clinical outcomes post treatment. To address this limitation, a single-agent group would be required in future trials.

Among the features that promote resistance to ICIs, expression of alternative immune checkpoints, lack of sufficient tumor antigens and presentation, and immune suppression by myeloid cells can all contribute,[39,40] and treatment with immune checkpoint therapy has been associated with an increase in MDSC and TAM populations, leading to secondary resistance to therapy.[21] Despite the lack of association of CEBPA upregulation with clinical outcome, changes to biological signals previously associated with MTL-CEBPA mode of action[20] were enriched in patients who showed disease stabilization. In TIMEPOINT, paired biopsy analysis suggests decreased immune suppression in both the circulation and in the TME, demonstrated by reduced MDSC populations and associated gene expression profiles after treatment. The reduction of immunosuppressive myeloid populations and increases in pro-inflammatory infiltrates demonstrated significance or a non-significant trend only in patients with disease stabilization, suggesting a link between these biological changes and treatment outcomes. These changes to immunosuppressive myeloid cells are consistent with previous observations in OUTREACH, a clinical trial of MTL-CEBPA in combination with sorafenib, suggesting that they may be specifically linked to MTL-CEBPA mode of action. However, in TIMEPOINT, the effects of pembrolizumab alone cannot be discounted or properly assessed in these data, and a monotherapy anti-PD-1 group would be required to do so, which was not possible in this phase I clinical trial.[4]

The concept of precision medicine is largely based on the application of biological-based indicators that can enrich for clinical response, with characteristics relating to the mode of action of the treatment representing promising biomarkers. MTL-CEBPA is proposed to act as a myeloid-targeting TME modulator, with primary action to reduce immunosuppressive myeloid cells in the TME.[20] In TIMEPOINT, patients with greater baseline proportions of immunosuppressive TAMs are more likely to show disease stabilization after treatment, linking to MTL-CEBPA mode of action. Furthermore, we also observed a relationship between circulating proteins and clinical outcome in the TIMEPOINT cohort. Interestingly, of the six proteins differentially expressed between patients with PD and those with disease stabilization in TIMEPOINT, three of the same proteins showed trend differences between clinical responders and non-responders in OUTREACH, indicating that these proteins as biological signals may be linked to MTL-CEBPA, rather than with pembrolizumab or sorafenib combination.

Furthermore, gene expression signatures based on tumor biopsies have been developed as predictive biomarkers, with the Oncotype Dx assay among the most well known.[41,42] In TIMEPOINT, we identified a transcriptional signature, CCGS, which was associated with clinical outcome in a univariable regression analysis in patients pre-treatment that had non-PD. Several of the genes related to myeloid-driven immunosuppression genes, supporting the mechanism of action of MTL-CEBPA. Consistent with this, CCGS appears to be associated with disease stabilization specifically post treatment with the combination of MTL-CEBPA and pembrolizumab, as traditional markers of anti-PD-1 response, such as T cell enrichment, PD1+ myeloid cells, and PD-L1 status,[29–32,43] were neither enriched in the CCGS signature nor did they associate with clinical outcome on their own. Further, the CCGS signature did not associate with clinical outcome in a melanoma population treated with pembrolizumab. Nevertheless, we cannot discount the possibility that CCGS is non-specific to the combination of MTL-CEBPA and pembrolizumab, and monotherapy groups would be required to further elucidate this. Additionally, given the heterogeneity of the patient population in this study and low patient numbers, substantial work would be needed to prospectively evaluate the predictive ability of this transcriptomic profile, to help identify subgroups of patients more likely to respond to combination treatment with MTL-CEBPA.

Limitations of the study

Our study has several limitations; first, TIMEPOINT enrolled a diverse study population, with very advanced disease that could have confounded the clinical and biological analyses. For example, the tumors came from many different primary organs, each with their own distinct physiological and biological ecosystem—not all the tumors have high levels of immune-suppressive myeloid cells or are PD-L1 positive, and most will have had divergence in the somatic mutation type and burden, extracellular matrix composition, and vasculature network. Though we included demographic data such as gender, ethnicity, and cancer type in the regression analyses with clinical outcome, accounting for the effect of demographic data and patient subgroups was not possible throughout the exploratory biological analyses due to the low number of patients included in the analyzed group from this phase 1 trial. Further to this, a second limitation is that our observations on the immunomodulatory effect of MTL-CEBPA on the TME, while informative, were based on the evaluation of small cohorts of patients (n = 23–24). The paired nature of these biopsies may offset some of that limitation. Additionally, given the relatively low occurrence of objective responses in this phase 1 clinical trial, we are largely unable to draw any firm conclusions regarding how changes observed in cytotoxic CD8 T cells, immunosuppressive myeloid cells, or inflammatory myeloid cells translate to better clinical outcome. We also note that future clinical success may be enhanced by the omission of steroids, as this compound class is now considered to suppress anti-tumor immunity, potentially limiting the efficacy of checkpoint inhibitors and other immune-activating agents.[44–47] Finally, as this trial is a combination of both MTL-CEBPA with pembrolizumab, it is not possible to isolate the singular effects of MTL-CEBPA versus pembrolizumab on the pharmacological changes we observed. We replicated several of the biological changes seen in a previous MTL-CEBPA combination trial, OUTREACH, and validated that pembrolizumab biomarkers were not associated with disease stabilization unlike MTL-CEBPA-related variables. While supportive, these data do not unequivocally prove what effects were solely attributable to MTL-CEBPA and which were solely attributable to pembrolizumab.

RESOURCE AVAILABILITY

Lead contact

Further information and reagents for resources and reagents should be directed to and will be fulfilled by the lead contact, Nagy A. Habib, nagy.habib@imperial.ac.uk.

Materials availability

This study did not generate any new reagents.

Data and code availability

- Deidentified Nanostring gene expression data from patients' tumor biopsies pre- and post treatment will be made available on publication at https://www.ncbi.nlm.nih.gov/geo/ under accession number GEO: GSE233860. The OLINK Proteomics data have been deposited to the ProteomeXchange Consortium via the PRIDE partner repository with the dataset identifier, PXD060726. Additional deidentified patient data will be available upon publication after approval of proposal by the chief investigator. Any additional data requests will be reviewed by the lead contact and Institutional Review Board of the participating sites, and, after approval, applying researchers must sign a data transfer agreement with MiNA Therapeutics. Previously reported CEBPA mRNA [19,20] was reanalyzed in the context of clinical response. RNA-seq of pre-treatment gene expression in pembrolizumab-treated patients with melanoma is from publicly available dataset, GEO: GSE78220.[48]
- This paper does not report original code. All of the scripts for analysis and figure production were written using open-source R, version 3.6 or version 4.2 and will be available on request in GitHub.
- Any additional information required to reanalyze the data reported in this paper is available from the lead contact on request.

ACKNOWLEDGMENTS

This work was supported by Experimental Cancer Medicine Centres, NIHR Biomedical Research Centres, and NHS Trust Tissue Banks at Imperial College London, Newcastle University, Kings College London, University of Cambridge, University of Liverpool, University of Birmingham, University of Glasgow, The University of Manchester, and University College London. The UK sites receive support from Cancer Research UK and Department of Health or Chief Scientist's Office, Scotland, as Experimental Cancer Medicine Centres. Financial support for the study was also provided by NIHR Biomedical Research Centre awards. Funding for preclinical work and clinical work was provided by MiNA Therapeutics. We would like to acknowledge Dr. Ilian Tchakov, Alison Adderkin, and Dr. Julia Vassiliadou for their work in the organization of TIMEPOINT clinical operations. We would like to thank the participating patients and their families for volunteering to take part in the study and all co-investigators and clinical and nursing staff for the conduct of the study.

This study was funded by MiNA Therapeutics.

AUTHOR CONTRIBUTIONS

R.P., M.H.S., B.M.R., V.R., J.V., D. Sarker, R. Habib, and N.A.H. contributed to the conception and design of study. D. Sarker, R.P., V.R., N.R., B.M.R., J.V., L.S., R. Hodgson, J.J.R., J.P.N., and N.A.H. contributed to the development of methodology. R.P., M.H.S., T.M., D.J.P., D. Sarker, B.B., S.B., N.C., T.R.J.E., J.Y., C.E.C., D.L., A.E.-K., M.D., K.-W.H., M.P., D. Spalding, T.T.,

M.S.N., B.K., D.M., M.-S.S., M.G., D.A., A.A., D.Z., J.S., I.M., and N.A.H. contributed to the acquisition of data (acquired and managed patients, and or provided facilities, etc.). M.H.S., B.M.R., R. Hodgson, N.R., V.R., J.V., L.S., A.A., J.J.R., and N.A.H. contributed to the analysis and interpretation of the data. M.-S.S., B.M.R., R. Hodgson, J.P.N., N.R., V.R., J.V., L.S., A.A., R. Habib, and N.A.H. contributed to the writing, review, or revision of the manuscript. J.P.N. contributed to the administrative support including reporting and organizing data and databases. R.P., M.H.S., T.M., D.J.P., D. Sarker, B.B., S.B., N.C., T.R.J.E., J.Y., C.E.C., D.L., A.E.-K., M.D., K.-W.H., M.P., D. Spalding, T.T., M.S.N., B.K., D.M., M.-S.S., D.Z., J.S., I.M., and N.A.H. contributed to study supervision. All authors contributed to (1) drafting the work or revising it critically for important intellectual content, (2) final approval of the version to be published, and (3) agreement to be accountable for all aspects of the work in ensuring that questions related to the accuracy or integrity of any part of the work are appropriately investigated and resolved.

DECLARATIONS OF INTERESTS

The following individuals are employees and shareholders of MiNA Therapeutics Ltd.: R. Habib, V.R., J.V., J.P.N., and N.A.H.

M.H.S. has a research grant from MiNA Therapeutics Ltd.

N.A.H. is the founder and director, and consults and advises at MiNA Therapeutics Ltd. and received travel, accommodation, and conference expenses support from MiNA Therapeutics Ltd. and institutional grant support from MiNA Therapeutics Ltd.

A.E.-K. has received research support from Astex, has received personal fees from Merrimack, and has served as an advisor for Bristol Myers Squibb, AstraZeneca, Bayer, Genentech, and Novartis.

T.M. has consultancies at Roche, AstraZeneca, Signant Health, GreyWolf, Guerbet, Geneos, Eisai, BeiGene, and MSD and received research funding from MSD, Bayer, and Boston Scientific.

D.J.P. consults, advises, and is on the speakers' bureau for Eisai and Roche and consults and advises for AstraZeneca, Avamune, BeiGene, Starpharma, BenevolentAI, Ipsen, and Mursla. He is on the speakers' bureau and received grants from Bayer and Bristol Myers Squibb. He consults for Da Volterra, Exact, and Mina Therapeutics. He is on the speakers' bureau for Falk Foundation and ViiV Healthcare. He received grants from GlaxoSmithKline, BMS, and MSD. Institutional Affiliation: secondary affiliation at the Department of Translational Medicine (DIMET), Universita' del Piemonte Orientale "A. Avogadro," Novara, Italy.

D. Sarker reports previous travel, accommodation, and conference expenses support from MiNA Therapeutics and receives consultancy or honoraria fees from Ipsen, Bayer, MSD, Roche, Servier, AstraZeneca, Boehringer, AbbVie, Sirtex, AAA, Incyte, and Eisai.

B.K. has received consulting fees from Regeneron, travel funds from Genentech/Roche, and research funding (to institution) from MiNA Therapeutics Limited, Partner Therapeutics, Apexigen, Antengene, Innovative Cellular Therapeutics Co., AstraZeneca, Takeda, Regeneron, and Genentech/Roche.

D.M. has received research funding from Amgen, Merck, Oncolytics, and Rafael; is on the scientific advisory board for Actuate and Qurient, is on an advisory/speaker bureau for Amgen, BMS, Eisai, and Exelixis; and has received funding paid to their institution from AbbVie, Acepodia, Actuate Therapeutics, ADC Therapeutics, Amgen, AVEO, Bayer, Blueprint Medicines, BMS, BioNTech, Dialectic Therapeutics, Epizyme, Fujifilm, ImmuneSensor, Immune-Onc Therapeutics, Leap Therapeutics, Lycera Corp., Merck, Millennium, MiNA Alpha, NGM Biopharmaceuticals, Novartis, Oncolytics, Orano Med, Puma, Qurient, Rafael, Repare Therapeutics, Triumvira Immunologics, Vigeo Therapeutics, and Werewolf Therapeutics.

S.B. receives institutional research funding to conduct clinical trials (of which PI or CI) from NuCana PLC, UCB, BioNTech, Medannex, Nurix, Theolytics, and Regeneron; consults for Ellipses, Oxford Investment Consultants, UCB, Simbec-Orion, and Theolytics; is Director at RNA Guardian Ltd.; and holds an ownership interest.

C.E.C. is an invited speaker at Amgen, AstraZeneca, Pierre Fabre, and Roche and is on the advisory board at Guardant Health AMEA, Merck, Pierre Fabre, and Roche.

I.M. is on advisory boards for AstraZeneca, GSK, Clovis Oncology, pharma&, BioNTech, and Roche.

T.R.J.E. declares honoraria (speaker's fees; advisory boards) from MSD payable to his employing institution; support for investigator-led clinical trials (MSD, pembrolizumab) payable to his employing institution; and support to attend international conferences (MSD) – personal and is Editor-in-Chief of the British Journal of Cancer.

M.S.N. receives research funding from Erytech and consults for Moderna and Merus.

N.C. has received research grants from Roche Pharmaceuticals, consulting fees from Roche Pharmaceuticals and Redx Pharmaceuticals, payment or honoraria for lectures, presentations, speakers bureaus, manuscript writing, or educational events from Roche Pharmaceuticals, support for attending meetings and/or travel from Roche Pharmaceuticals, and research funding to research team from AstraZeneca, Orion, F. Hoffmann-La Roche Ltd., Taiho, GSK, Novartis, Starpharma, Bayer, Eisai, UCB, RedX Pharmaceuticals, Stemline Therapeutics, LOXO-oncology, Avacta, Boehringer Ingelheim, Merck, and Tarveda Therapeutics and participated in data safety monitoring board or advisory board at Roche Pharmaceuticals and Cancer Research UK.

STAR★METHODS

Detailed methods are provided in the online version of this paper and include the following:

- KEY RESOURCES TABLE
- EXPERIMENTAL MODEL AND STUDY PARTICIPANT DETAILS
  - Patients
- METHOD DETAILS
  - Study design
  - Outcomes
  - OUTREACH clinical trial
  - Pharmacokinetics
  - RNA expression analysis
  - PMN-MDSC quantification from patient blood
  - Veracyte Brightplex technology: Sequential multiplex Immunohistochemistry (IHC)
  - *Veracyte Brightplex* technology: Digital *pathology analysis*
  - NanoString analysis
  - Differential expression analyses
  - OLINK
  - Protein detection of CEBPA in monocytes and lymphocytes
- QUANTIFICATION AND STATISTICAL ANALYSIS
  - Statistical analysis
- ADDITIONAL RESOURCES

SUPPLEMENTAL INFORMATION

Supplemental information can be found online at https://doi.org/10.1016/j.xcrm.2025.102041.

Received: November 16, 2023
Revised: March 5, 2024
Accepted: March 3, 2025
Published: March 31, 2025

REFERENCES

1. Hodi, F.S., O'Day, S.J., McDermott, D.F., Weber, R.W., Sosman, J.A., Haanen, J.B., Gonzalez, R., Robert, C., Schadendorf, D., Hassel, J.C., et al. (2010). Improved Survival with Ipilimumab in Patients with Metastatic Melanoma. N. Engl. J. Med. *363*, 711–723.
2. Robert, C., Ribas, A., Hamid, O., Daud, A., Wolchok, J.D., Joshua, A.M., Hwu, W.J., Weber, J.S., Gangadhar, T.C., Joseph, R.W., et al. (2018). Durable Complete Response After Discontinuation of Pembrolizumab in Patients With Metastatic Melanoma. J. Clin. Oncol. *36*, 1668–1674.

CellPress
OPEN ACCESS

3. Vaddepally, R.K., Kharel, P., Pandey, R., Garje, R., and Chandra, A.B. (2020). Review of Indications of FDA-Approved Immune Checkpoint Inhibitors per NCCN Guidelines with the Level of Evidence. Cancers *12*, 738.
4. Piha-Paul, S.A., Oh, D.Y., Ueno, M., Malka, D., Chung, H.C., Nagrial, A., Kelley, R.K., Ros, W., Italiano, A., Nakagawa, K., et al. (2020). Efficacy and safety of pembrolizumab for the treatment of advanced biliary cancer: Results from the KEYNOTE-158 and KEYNOTE-028 studies. Int. J. Cancer *147*, 2190–2198.
5. Bian, J., and Almhanna, K. (2021). Pancreatic cancer and immune checkpoint inhibitors—still a long way to go. Transl. Gastroenterol. Hepatol. *6*, 6.
6. Borella, F., Ghisoni, E., Giannone, G., Cosma, S., Benedetto, C., Valabrega, G., and Katsaros, D. (2020). Immune Checkpoint Inhibitors in Epithelial Ovarian Cancer: An Overview on Efficacy and Future Perspectives. Diagnostics *10*, 146.
7. Ueno, M., Chung, H., Nagrial, A., Marabelle, A., Kelley, R., Xu, L., Mahoney, J., Pruitt, S., and Oh, D.Y. (2018). Pembrolizumab for advanced biliary adenocarcinoma: Results from the multicohort, phase II KEYNOTE-158 study. Ann. Oncol. *29*, viii210.
8. Carpenter, E., Nelson, S., Bednar, F., Cho, C., Nathan, H., Sahai, V., di Magliano, M.P., and Frankel, T.L. (2021). Immunotherapy for pancreatic ductal adenocarcinoma. J. Surg. Oncol. *123*, 751–759.
9. Li, T., Liu, T., Zhu, W., Xie, S., Zhao, Z., Feng, B., Guo, H., and Yang, R. (2021). Targeting MDSC for Immune-Checkpoint Blockade in Cancer Immunotherapy: Current Progress and New Prospects. Clin. Med. Insights Oncol. *15*, 11795549211035540.
10. Veglia, F., Perego, M., and Gabrilovich, D. (2018). Myeloid-derived suppressor cells coming of age. Nat. Immunol. *19*, 108–119.
11. Sarker, D. Plummer, E.R., Basu, B., Meyer, T., Huang, K.W., Evans, T.J., Spalding, D., Ma, Y.T., Palmer, D.H., Chee, C.E. and Toh, H.C First-in-human, first-in-class phase I study of MTL-CEBPA, a small activating RNA (saRNA) targeting the transcription factor C/EBP-α in patients with advanced liver cancer. https://doi.org/10.1200/JCO.2017.35.15_suppl.TPS2612 35, TPS2612–TPS2612 (2017).
12. Bereshchenko, O., Mancini, E., Moore, S., Bilbao, D., Månsson, R., Luc, S., Grover, A., Jacobsen, S.E.W., Bryder, D., and Nerlov, C. (2009). Hematopoietic stem cell expansion precedes the generation of committed myeloid leukemia-initiating cells in C/EBPalpha mutant AML. Cancer Cell *16*, 390–400.
13. Kirstetter, P., Schuster, M.B., Bereshchenko, O., Moore, S., Dvinge, H., Kurz, E., Theilgaard-Mönch, K., Månsson, R., Pedersen, T.A., Pabst, T., et al. (2008). Modeling of C/EBPalpha mutant acute myeloid leukemia reveals a common expression signature of committed myeloid leukemia-initiating cells. Cancer Cell *13*, 299–310.
14. Pabst, T., Mueller, B.U., Zhang, P., Radomska, H.S., Narravula, S., Schnittger, S., Behre, G., Hiddemann, W., and Tenen, D.G. (2001). Dominant-negative mutations of CEBPA, encoding CCAAT/enhancer binding protein-alpha (C/EBPalpha), in acute myeloid leukemia. Nat. Genet. *27*, 263–270.
15. Porse, B.T., Bryder, D., Theilgaard-Mönch, K., Hasemann, M.S., Anderson, K., Damgaard, I., Jacobsen, S.E.W., and Nerlov, C. (2005). Loss of C/EBP alpha cell cycle control increases myeloid progenitor proliferation and transforms the neutrophil granulocyte lineage. J. Exp. Med. *202*, 85–96.
16. Zhang, D.E., Zhang, P., Wang, N.D., Hetherington, C.J., Darlington, G.J., and Tenen, D.G. (1997). Absence of granulocyte colony-stimulating factor signaling and neutrophil development in CCAAT enhancer binding protein alpha-deficient mice. Proc. Natl. Acad. Sci. USA *94*, 569–574.
17. Zhang, P., Iwasaki-Arai, J., Iwasaki, H., Fenyus, M.L., Dayaram, T., Owens, B.M., Shigematsu, H., Levantini, E., Huettner, C.S., Lekstrom-Himes, J.A., et al. (2004). Enhancement of hematopoietic stem cell repopulating capacity and self-renewal in the absence of the transcription factor C/EBP alpha. Immunity *21*, 853–863.
18. Sarker, D., Sodergren, M., Plummer, E.R., Basu, B., Meyer, T., Huang, K.W., Evans, T.J., Spalding, D., Ma, Y.T., Palmer, D.H., et al., Phase Ib dose escalation and cohort expansion study of the novel myeloid differentiating agent MTL-CEBPA in combination with sorafenib in patients with advanced hepatocellular carcinoma (HCC). https://doi.org/10.1200/JCO.2020.38.15_suppl.4601 38, 4601 (2020).38,
19. Sarker, D., Plummer, R., Meyer, T., Sodergren, M.H., Basu, B., Chee, C.E., Huang, K.W., Palmer, D.H., Ma, Y.T., Evans, T.R.J., et al. (2020). MTL-CEBPA, a Small Activating RNA Therapeutic Upregulating C/EBP-α, in Patients with Advanced Liver Cancer: A First-in-Human, Multicenter, Open-Label, Phase I Trial. Clin. Cancer Res. *26*, 3936–3946.
20. Hashimoto, A., Sarker, D., Reebye, V., Jarvis, S., Sodergren, M.H., Kossenkov, A., Sanseviero, E., Raulf, N., Vasara, J., Andrikakou, P., et al. (2021). Upregulation of C/EBPα inhibits suppressive activity of myeloid cells and potentiates antitumor response in mice and patients with cancer. Clin. Cancer Res. *27*, 5961–5978.
21. Sun, S.H., Benner, B., Savardekar, H., Lapurga, G., Good, L., Abood, D., Nagle, E., Duggan, M., Stiff, A., DiVincenzo, M.J., et al. (2021). Effect of Immune Checkpoint Blockade on Myeloid-Derived Suppressor Cell Populations in Patients With Melanoma. Front. Immunol. *12*, 740890.
22. Peranzoni, E., Ingangi, V., Masetto, E., Pinton, L., and Marigo, I. (2020). Myeloid Cells as Clinical Biomarkers for Immune Checkpoint Blockade. Front. Immunol. *11*, 1590.
23. Scholler, N., Perbost, R., Locke, F.L., Jain, M.D., Turcan, S., Danan, C., Chang, E.C., Neelapu, S.S., Miklos, D.B., Jacobson, C.A., et al. (2022). Tumor immune contexture is a determinant of anti-CD19 CAR T cell efficacy in large B cell lymphoma. Nat. Med. *28*, 1872–1882.
24. Garner, H., and de Visser, K.E. (2020). Immune crosstalk in cancer progression and metastatic spread: a complex conversation. Nat. Rev. Immunol. *20*, 483–497.
25. Gabrilovich, D.I., and Nagaraj, S. (2009). Myeloid-derived-suppressor cells as regulators of the immune system. Nat. Rev. Immunol. *9*, 162–174.
26. Chen, D.S., and Mellman, I. (2013). Oncology meets immunology: The cancer-immunity cycle. Immunity *39*, 1–10.
27. Scholler, N., Perbost, R., Locke, F.L., Jain, M.D., Turcan, S., Danan, C., Chang, E.C., Neelapu, S.S., Miklos, D.B., Jacobson, C.A., et al. (2022). Tumor immune contexture is a determinant of anti-CD19 CAR T cell efficacy in large B cell lymphoma. Nat. Med. *28*, 1872–1882.
28. Galon, J., Rossi, J., Turcan, S., Danan, C., Locke, F.L., Neelapu, S.S., Miklos, D.B., Bartlett, N.L., Jacobson, C.A., Braunschweig, I., et al. (2017). Characterization of anti-CD19 chimeric antigen receptor (CAR) T cell-mediated tumor microenvironment immune gene profile in a multicenter trial (ZUMA-1) with axicabtagene ciloleucel (axi-cel, KTE-C19). J. Clin. Oncol. *35*, 3025.
29. Cristescu, R., Mogg, R., Ayers, M., Albright, A., Murphy, E., Yearley, J., Sher, X., Liu, X.Q., Lu, H., Nebozhyn, M., et al. (2018). Pan-tumor genomic biomarkers for PD-1 checkpoint blockade–based immunotherapy. Science *362*, eaar3593.
30. Ayers, M., Lunceford, J., Nebozhyn, M., Murphy, E., Loboda, A., Kaufman, D.R., Albright, A., Cheng, J.D., Kang, S.P., Shankaran, V., et al. (2017). IFN-γ–related mRNA profile predicts clinical response to PD-1 blockade. J. Clin. Investig. *127*, 2930–2940.
31. Liu, Y., Zugazagoitia, J., Ahmed, F.S., Henick, B.S., Gettinger, S.N., Herbst, R.S., Schalper, K.A., and Rimm, D.L. (2020). Immune Cell PD-L1 Colocalizes with Macrophages and Is Associated with Outcome in PD-1 Pathway Blockade Therapy. Clin. Cancer Res. *26*, 970–977.
32. Ahn, S., Lee, J. chan, Shin, D.W., Kim, J., and Hwang, J.H. (2020). High PD-L1 expression is associated with therapeutic response to pembrolizumab in patients with advanced biliary tract cancer. Sci. Rep. *10*, 12348.
33. Zhang, J., Huang, D., Saw, P.E., and Song, E. (2022). Turning cold tumors hot: from molecular mechanisms to clinical applications. Trends Immunol. *43*, 523–545.
34. Tumeh, P.C., Harview, C.L., Yearley, J.H., Shintaku, I.P., Taylor, E.J.M., Robert, L., Chmielowski, B., Spasic, M., Henry, G., Ciobanu, V., et al.

(2014). PD-1 blockade induces responses by inhibiting adaptive immune resistance. Nature *515*, 568–571.

35. Gattinoni, L., Speiser, D.E., Lichterfeld, M., and Bonini, C. (2017). T memory stem cells in health and disease. Nat. Med. *23*, 18–27.

36. Sabatino, M., Hu, J., Sommariva, M., Gautam, S., Fellowes, V., Hocker, J.D., Dougherty, S., Qin, H., Klebanoff, C.A., Fry, T.J., et al. (2016). Generation of clinical-grade CD19-specific CAR-modified CD8+ memory stem cells for the treatment of human B-cell malignancies. Blood *128*, 519–528.

37. Kilian, M., Sheinin, R., Tan, C.L., Friedrich, M., Krämer, C., Kaminitz, A., Sanghvi, K., Lindner, K., Chih, Y.C., Cichon, F., et al. (2023). MHC class II-restricted antigen presentation is required to prevent dysfunction of cytotoxic T cells by blood-borne myeloids in brain tumors. Cancer Cell *41*, 235–251. https://doi.org/10.1016/J.CCELL.2022.12.007.

38. Prokhnevska, N., Cardenas, M.A., Valanparambil, R.M., Sobierajska, E., Barwick, B.G., Jansen, C., Reyes Moon, A., Gregorova, P., delBalzo, L., Greenwald, R., et al. (2023). CD8+ T cell activation in cancer comprises an initial activation phase in lymph nodes followed by effector differentiation within the tumor. Immunity *56*, 107–124.

39. Bagchi, S., Yuan, R., and Engleman, E.G. (2021). Immune Checkpoint Inhibitors for the Treatment of Cancer: Clinical Impact and Mechanisms of Response and Resistance. Annu. Rev. Pathol. *16*, 223–249.

40. Jenkins, R.W., Barbie, D.A., and Flaherty, K.T. (2018). Mechanisms of resistance to immune checkpoint inhibitors. Br. J. Cancer *118*, 9–16.

41. McVeigh, T.P., and Kerin, M.J. (2017). Clinical use of the Oncotype DX genomic test to guide treatment decisions for patients with invasive breast cancer. Breast Cancer *9*, 393–400.

42. Sparano, J.A., and Paik, S. (2008). Development of the 21-gene assay and its application in clinical practice and clinical trials. J. Clin. Oncol. *26*, 721–728.

43. Kim, M.J., and Ha, S.J. (2021). Differential Role of PD-1 Expressed by Various Immune and Tumor Cells in the Tumor Immune Microenvironment: Expression, Function, Therapeutic Efficacy, and Resistance to Cancer Immunotherapy. Front. Cell Dev. Biol. *9*, 767466.

44. Kalfeist, L., Galland, L., Ledys, F., Ghiringhelli, F., Limagne, E., and Ladoire, S. (2022). Impact of Glucocorticoid Use in Oncology in the Immunotherapy Era. Cells *11*, 770.

45. Goodman, R.S., Johnson, D.B., and Balko, J.M. (2023). Corticosteroids and Cancer Immunotherapy. Clin. Cancer Res. *29*, 2580–2587. https://doi.org/10.1158/1078-0432.CCR-22-3181.

46. Pant, A., Medikonda, R., and Lim, M. (2020). Alternative Checkpoints as Targets for Immunotherapy. Curr. Oncol. Rep. *22*, 126.

47. Loch, F.N., Kamphues, C., Beyer, K., Schineis, C., Rayya, W., Lauscher, J.C., Horst, D., Dragomir, M.P., and Schallenberg, S. (2023). The Immune Checkpoint Landscape in Tumor Cells of Pancreatic Ductal Adenocarcinoma. Int. J. Mol. Sci. *24*, 2160.

48. Hugo, W., Zaretsky, J.M., Sun, L., Song, C., Moreno, B.H., Hu-Lieskovan, S., Berent-Maoz, B., Pang, J., Chmielowski, B., Cherry, G., et al. (2016). Genomic and Transcriptomic Features of Response to Anti-PD-1 Therapy in Metastatic Melanoma. Cell *165*, 35–44.

49. Voutila, J., Reebye, V., Roberts, T.C., Protopapa, P., Andrikakou, P., Blakey, D.C., Habib, R., Huber, H., Saetrom, P., Rossi, J.J., and Habib, N.A. (2017). Development and Mechanism of Small Activating RNA Targeting CEBPA, a Novel Therapeutic in Clinical Trials for Liver Cancer. Mol. Ther. *25*, 2705–2714.

50. Kassambara, A. (2021). rstatix: Pipe-Friendly Framework for Basic Statistical Tests. R package version 0.7.2. https://rpkgs.datanovia.com/rstatix/.

51. Wickham, H. ggplot2: Elegant Graphics for Data Analysis. https://ggplot2.tidyverse.org Preprint at (2016).

52. Blighe, K., Rana S & Lewis M. EnhancedVolcano: Publication-ready volcano plots with enhanced colouring and labeling. https://github.com/kevinblighe/EnhancedVolcano Preprint at (2024).

53. Wickham, H., François, R., Henry, L., Müller, K., and Vaughan, D. (2023). dplyr: A Grammar of Data Manipulation. R package version 1.1.4. https://github.com/tidyverse/dplyr.

54. Love, M.I., Huber, W., and Anders, S. (2014). Moderated estimation of fold change and dispersion for RNA-seq data with DESeq2. Genome Biol. *15*, 550.

55. Huber, W., von Heydebreck, A., Sültmann, H., Poustka, A., and Vingron, M. (2002). Variance stabilization applied to microarray data calibration and to the quantification of differential expression. Bioinformatics *18*, S96–S104.

56. Kassambara, A. ggpubr: 'ggplot2' Based Publication Ready Plots. https://rpkgs.datanovia.com/ggpubr/ Preprint at (2023).

57. Patil, I. (2021). Visualizations with statistical details: The 'ggstatsplot' approach. J. Open Source Softw. *6*, 3167.

58. Kassambara, A., Kosinski, M. & Biecek, P. survminer: Survival Analysis and Visualization.2019. https://rpkgs.datanovia.com/survminer/index.html.

59. Therneau, T. M. A Package for Survival Analysis in R.2004. https://CRAN.R-project.org/package=survival.

60. Korotkevich, G., Sukhov, V., and Sergushichev, A. (2019). Fast gene set enrichment analysis. bioRxiv.

61. Risso, D., Ngai, J., Speed, T.P., and Dudoit, S. (2014). Normalization of RNA-seq data using factor analysis of control genes or samples. Nat. Biotechnol. *32*, 896–902.

62. Guangchuang, Y. (2021). Visualization of Functional Enrichment Result. R package version *1.24.0*. https://yulab-smu.top/biomedical-knowledge-mining-book/.

63. Anders, S., and Huber, W. (2010). Differential expression analysis for sequence count data. Genome Biol. *11*, R106.

64. Bhattacharya, A., Hamilton, A.M., Furberg, H., Pietzak, E., Purdue, M.P., Troester, M.A., Hoadley, K.A., and Love, M.I. (2021). An approach for normalization and quality control for NanoString RNA expression data. Briefings Bioinf. *22*, bbaa163.

65. Damotte, D., Warren, S., Arrondeau, J., Boudou-Rouquette, P., Mansuet-Lupo, A., Biton, J., Ouakrim, H., Alifano, M., Gervais, C., Bellesoeur, A., et al. (2019). The tumor inflammation signature (TIS) is associated with anti-PD-1 treatment benefit in the CERTIM pan-cancer cohort. J. Transl. Med. *17*, 357.

66. Lim, G.B., Kim, Y.A., Seo, J.H., Lee, H.J., Gong, G., and Park, S.H. (2020). Prediction of prognostic signatures in triple-negative breast cancer based on the differential expression analysis via NanoString nCounter immune panel. BMC Cancer *20*, 1052.

67. Patel, H., and Davidson, D. (2014). Control of pro-inflammatory cytokine release from human monocytes with the use of an interleukin-10 monoclonal antibody. Methods Mol. Biol. *1172*, 99–106.

STAR★METHODS

KEY RESOURCES TABLE

REAGENT or RESOURCE	SOURCE	IDENTIFIER
Antibodies		
FITC-conjugated CD11b Antibody, anti-human, REAfinity	Miltenyi Biotec	Catalog #130-110-552, clone REA713
APC-Vio770-conjugated CD14 Antibody, anti-human, REAfinity	Miltenyi Biotec	Catalog #130-110-522, clone REA599
PerCP-Vio700-conjugated CD15 Antibody, anti-human	Miltenyi Biotec	Catalog #130-113-487, clone VIMC6
PE anti-human LOX-1 Antibody	Biolegend	Catalog #358604, clone 15C4
APC-conjugated HLA-DR Antibody, anti-human, REAfinity	Miltenyi Biotec	Catalog #130-111-790 clone REA805
BD Horizon™ V500 Mouse Anti-Human CD3	BD Biosciences	Catalog #561416, clone UCHT-1
CD56 (NCAM) Monoclonal Antibody (TULY56), eFluor™ 506	eBioscience	Catalog #15520677, clone TULY56
BD Horizon™ V500 Mouse anti-Human CD19	BD Biosciences	Catalog #561121 clone HIB19
Invitrogen™ eBioscience™ Fixable Viability Dye eFluor™ 506	eBioscience	Catalog #65-0866-14
StainExpress™ Immune Cell Composition Cocktail kit	Miltenyi Biotec	Catalog #130-127-637
Anti-human LOX-1 Antibody	R&D Systems	Catalog #MABS186
BD Pharmingen™ Purified Mouse Anti-Human CD15	BD Biosciences	Catalog #555400, clone HI98
Anti-human CD11b/ITGAM (D6X1N) Rabbit mAb	Cell Signaling Technologies	Catalog #49420S, clone D6X1N
Anti-human CD14 antibody	Sigma	Catalog #114R-15, clone EPR3653
Anti-human S100A9 Monoclonal Antibody (UMAB173), UltraMAB	Origene	Catalog #UM800066, clone UMAB173
Anti-human HLA-DR Antibody (TAL 1B5)	Santa Cruz	Catalog # Sc-53319, clone TAL1B5
Anti-human CD3 Antibody	Halio DX	Reference: HD-FG-000013
Anti-human CD8 Antibody	Halio DX	Reference: HD-FG-000019
Anti-human PDL1 Antibody	Halio DX	Reference: HD-FG-000035
Anti-human Ki-67 Antibody	Cell Signaling Technologies	Catalog # 9027, clone D2H10
Anti-human Granzyme B Antibody	Dako	Catalog #M72350
Anti-human PD1 (PDCD1) Mouse Monoclonal Antibody	Origene	Catalog # UM800091, clone UMAB199
Anti-human CD68 Antibody	Abcam	Catalog #ab213363, clone EPR20545
Anti-human CD163 Antibody	Abcam	Catalog #ab182422, clone EPR195198
Anti-human CD64 Antibody	Abcam	Catalog # ab140779, clone OTI3D3
Mouse MMR/CD206 Antibody	R & D Systems	Catalog # MAB25341
MACH 2 Rabbit HRP-Polymer	Biocare	Catalog # RHRP520
MACH 4 Universal HRP polymer	Biocare	Catalog #M4U534L
Anti-human anti-CEBPA primary antibody	Cell Signaling Technology	Catalog # 8178, clone D56F10
Biological samples		
Patient derived blood samples	On study	TIME POINT Clinical trial; NCT04105335
Patient derived white blood cell (WBC) samples	On study	TIME POINT Clinical trial; NCT04105335
Patient derived serum samples	On study	TIME POINT Clinical trial; NCT04105335
Patient derived plasma samples	On study	TIME POINT Clinical trial; NCT04105335
Patient derived tumor biopsies	On study	TIME POINT Clinical trial; NCT04105335

(Continued on next page)

Continued		
REAGENT or RESOURCE	SOURCE	IDENTIFIER
Patient derived RNA from WBC samples	On study[19,20]	OUTREACH Clinical trial; NCT02716012
Patient derived plasma samples	On study	OUTREACH Clinical trial; NCT02716012
Critical commercial assays		
QIAGEN RNeasy FFPE extraction kits	Qiagen	Catalog #73504
Nanostring PanCancer IO360 Profiling Panel	Nanostring	Nanostring
OLINK Proximity extension assay - Target 96 Inflammation panel	OLINK	The Leeds Immunogenomics Facility
Veracyte Brightplex® technology immunohistochemistry using MDSC Panel, T Cells Effector Panel, Macrophage Panel	Veracyte	Veracyte, Paris
CEBPA primer		QIAGEN: assay ID: Hs00269972_s1
GAPDH primer	Quantitect primer assay	QIAGEN: assay ID: Hs99999905_m1
Deposited data		
Nanostring-derived gene expression data from tumor biopsies pre- and post-MTL-CEBPA and pembrolizumab therapy	This paper	GEO: GSE233860
OLINK Affinity proteomics data from plasma of patients before treatment with MTL-CEBPA and combination treatments. These data are associated with TIME POINT and OUTREACH clinical trials, NCT04105335 and NCT02716012.	This paper	PXD060726
mRNA expressions in pre-treatment melanomas undergoing anti-PD-1 checkpoint inhibition therapy	PMID: 26997480	GEO: GSE78220
Oligonucleotides		
MTL-CEBPA	MiNA Therapeutics, Voutila, J. et al.[49]	MiNA Therapeutics, Voutila, J. et al.[49]
Software and algorithms		
R version 4.2.0	https://cran.r-project.org/bin/windows/base/	CRAN
Rstudio	https://posit.co/	Rstudio-desktop
R package, Rstatix	Kassambara, A.[50]	CRAN or Bioconductor
R package, ggplot2	Wickham, H.[51]	CRAN or Bioconductor
R package, EnhancedVolcano	Blighe, K. et al.[52]	CRAN or Bioconductor
R package, dplyr	Wickham, H. et al.[53]	CRAN or Bioconductor
R package, DESeq2	Love, M. et al.[54]	CRAN or Bioconductor
R package, vsn	Huber, W. et al.[55]	CRAN or Bioconductor
R package, ggpubr	Kassambara, A.[56]	CRAN or Bioconductor
R package, ggstatsplot	Patil, I.[57]	CRAN or Bioconductor
R package, survminer	Kassambara, A. et al.[58]	CRAN or Bioconductor
R package, survival	Therneau, T. M.[59]	CRAN or Bioconductor
R package, fgsea	Korotkevich G. et al.[60]	CRAN or Bioconductor
R package, RUVSeq	Risso, D. et al.[61]	CRAN or Bioconductor
R package, enrichplot	Guangchuang, Y.[62]	CRAN or Bioconductor
Brightplex HalioDx technology	Veracyte	Veracyte
GraphPad Prism 9 (version 9.1.2 (225) or above)	GraphPad	GraphPad Software, Inc., San Diego, CA
FlowJo Software	FlowJo	https://www.flowjo.com/

(*Continued on next page*)

Continued

REAGENT or RESOURCE	SOURCE	IDENTIFIER
Compass Software (ProteinSimple)	Biotechne	Biotechne
Phoenix WinNonlin version 7.0	Certara	Certara

EXPERIMENTAL MODEL AND STUDY PARTICIPANT DETAILS

Patients

TIME POINT clinical trial is titled ‘‘AN OPEN LABEL PHASE 1A/B STUDY OF MTL-CEBPA IN COMBINATION WITH A PD-1 INHIBITOR (PEMBROLIZUMAB) IN ADULT PATIENTS WITH ADVANCED SOLID TUMORS’’, Clinicaltrials.gov number: NCT04105335. The study was submitted and approved by the UK competent Authority (MHRA) on 10 July 2019 (CTA 45697/0002/001-0001), UK REC on 13 November 2019 (North East- Newcastle & North Tyneside 2) and Health Research Authority (HRA) 23 October 2019. Subsequent approvals to open studies in non-UK countries were granted by competent authorities and ethics committees in France, Sweden, Denmark, USA, Singapore, Taiwan.

All patients signed a country specific consent form prior to screening visit which included permission to analyze historic tumor samples, take blood for research and store blood/serum and tissue in an accredited tissue bank for future research.

We report results (data cut-off 15 March 2022 for planned interim biomarker analysis with no further recruitment to date and until completion of analysis) from an ongoing multi-centre, non-comparative, open-label, phase 1 study in patients with any solid tumor whose disease progressed on standard of care therapy or for whom no therapy was available. According to the protocol, this study intended to recruit 108 patients, and we now report on a pre-specified interim biomarker analysis of 50 patients. This study was conducted at 13 tertiary centers and university hospitals. Eligible patients were at least 18 years old with histologically or cytologically confirmed diagnosis of any solid tumor. Patients were required to be naive to anti-PD-1/PD-L1 antibodies, Eastern Cooperative Oncology Group score (ECOG) performance status 0–1 and with a life expectancy greater than 3 months at the time of recruitment. Information on patient gender, ethnicity, age, health status and previous lines of therapy can be found in Table S2. All patients provided written informed consent, and the study protocol, MNA-3521-012; IRAS ID: 266862; EudraCT 2019-002231-28 and all amendments were approved by the North East - Newcastle & North Tyneside 2 Research Ethics Committee, approval number 19/NE/0312 and each site’s institutional review board or independent ethics committee. Gender, ethnicity and trial phase were included within the univariable cox regression analysis, however were not included in other analyses throughout the manuscript due to the low patient numbers available in many of the assays (<25). The absence of these analyses limits the generalisability of the study.

METHOD DETAILS

Study design

The study comprised a dose escalation of three planned cohorts in a 3 + 3 design in which doses of MTL-CEBPA were escalated in sequential cohorts at the following dose levels: 70 mg/m^2 dosing frequency once per week (QW), 98 mg/m^2 QW, and 130 mg/m^2 QW and combined with standard dose of pembrolizumab (200mg) in all patients given every 3 weeks followed by dose expansion at Recommended Phase 2 dose (RP2D).

MTL-CEBPA was administered by intravenous infusion over 60 min once a week for 3 weeks followed by a rest period of 1 week; this defines a 4-week cycle. MTL-CEBPA dosing was preceded by prednisolone/hydrocortisone and anti-histamine administration to minimise the risk of infusion reactions. The determination of the starting dose of MTL-CEBPA was based on our previously reported Phase 1 study of MTL-CEBPA for HCC in which a maximum weekly dose of 210 mg/m^2 (70 mg/m^2 TIW three times per week) was administered with no dose-limiting toxicity (DLT) confirmed and no maximum tolerated dose (MTD) reached. The RP2D from this study was determined at 130 mg/m^2 weekly as both monotherapy and in combination with sorafenib. One of the aims of this study was to support the MTL-CEBPA RP2D determination and dose optimisation and confirm dose as tolerable in combination with pembrolizumab. We therefore considered a dose level of 70 mg/m^2 QW as a safe and appropriate starting dose for MTL-CEBPA in this study.

Pembrolizumab (200mg) was administered on Day 2 of the first cycle and subsequently every 3 weeks whilst the patients is on treatment.

The DLT were determined on the basis of a standard 3 + 3 dose escalation design with the incidence and severity of adverse events (AEs) occurring in the first cycle (28 days). A Safety Review Committee (SRC) was convened to oversee safety, scientific integrity and validity of the study. All dose escalation decisions were made by the SRC. Patients were treated until disease progression, unacceptable toxicity, withdrawal of consent or death. Patients remained in follow-up post-discontinuation of study drug to assess for overall survival (OS). Safety and tolerability of MTL-CEBPA was evaluated in terms of frequency of AEs graded according to toxicity criteria (NCI Common Terminology Criteria for Adverse Events, CTCAE v 5.0). Tumor response and progression was evaluated using the revised Response Evaluation Criteria in Solid Tumors (RECIST) guideline version 1.1 and modified RECIST (mRECIST) from the completion of Cycle 2. The study was conducted with blinded independent central radiology review in place. There was high concordance in the assessment of best objective response between local and the blinded independent central radiology review.

Outcomes

The primary endpoint in this study is to determine the safety and tolerability of MTL-CEBPA in combination with pembrolizumab (Part 1a) and the anti-tumour activity of the combination as assessed by objective response (ORR) (Part 1b). The DLT was defined as any drug related toxicity grade ≥3 according to the CTCAE with the only exception of aspartate transaminase (AST)/alanine transaminase (ALT) related DLT defined as Grade 4 AST and/or ALT abnormal laboratory value > 20.0 x upper limit of normal (ULN). Secondary endpoints included the determination of RP2D and incidence of toxicity as measured by AEs, serious adverse events (SAEs) and laboratory tests graded according to toxicity criteria (CTCAE) characterisation of pharmacokinetics (PK), pharmacodynamics (PD) and anti-tumour activity.

OUTREACH clinical trial

This manuscript also reports proteomic data from patients treated with MTL-CEBPA in combination with sorafenib from clinical trial, OUTREACH, accessible at ClinicalTrials.gov, number NCT02716012 and reported.[19] Stored plasma from patients from this clinical trial were analyzed using OLINK as described below. Additionally, RNA from this study was reanalysed in the context of clinical response and represented here.

Pharmacokinetics

CEBPA-51 is a 21mer saRNA duplex oligonucleotide and is detected in plasma by extraction and dissociation of the duplex from MTL-CEBPA nanoparticles. Due to the rapid degradation and elimination of free CEBPA-51 (the active pharmaceutical ingredient) in plasma, measurement of CEBPA-51 oligo concentration reflects the concentration of CEBPA-51 encapsulated in intact MTL-CEBPA nanoparticles. A fluorescently labeled peptide nucleic acid (PNA)-probe, designed against the guide strand of CEBPA-51, was used to extract the single-stranded parent compound. RNA species were then quantitated using anion-exchange high-performance liquid chromatography (HPLC) and fluorescence detection. Plasma CEBPA-51 is expressed as μg/mL of double-stranded RNA and the lower limit of quantitation is 0.001 μg/mL. Plasma samples for analysis of CEBPA-51 were collected over the first dosing interval for each Q1wk regimen and for 72h after administration of the second dose.

RNA expression analysis

Blood samples were collected at pre-treatment (before start of infusion at Cycle 1 Day 1; C1D1) and then 24 h after initial treatment (C1 D2). Ten millilitres of blood were collected in EDTA vacutainers (BD Pharmigen), captured in a LeukoLOCK filter system (Ambion) and used for RNA extraction and cDNA synthesis as described previously.[19,49] Transcript levels for CEBPA were measured by quantitative PCR (qPCR) (QuantStudio 5) using TaqMan gene expression assay. Quantitect Primer Assays (QIAGEN) were used: CEBPA (assay ID:Hs00269972_s1) and GAPDH (assay ID: Hs99999905_m1). GAPDH served as housekeeping gene.

PMN-MDSC quantification from patient blood

Whole blood (5–8 mL) was collected from trial subjects in Streck CytoChex vacutainers at D0 (pre-treatment), 24 h after first infusion (D1), 48 h after first infusion (D3) of MTL-CEBPA. The blood was processed within 120 min of collection. Briefly, 100 μL whole blood was added to an antibody cocktail, prepared in 50ul Cell staining buffer (BD Bioscience #42020). For MDSCs, this cocktail includes CD11b FITC (Miltenyi 130-110-552, clone REA713), CD14 APC-Vio770 (Miltenyi 130-110-522, clone REA599), CD15 PerCP-Vio700 (Miltenyi 130-113-487, clone VIMC6), LOX-1 PE (BioLegend 358604, clone 15C4), HLA-DR APC (Miltenyi 130-111-790 clone REA805), lineage markers: CD3 V500 (BD Horizon 561416, clone UCHT1), CD56 eFluor506 (eBiosciences 15520677, clone TULY56), CD19 V500 (BD Horizon 561121 clone HIB19), Dead cell exclusion dye eFluor 506 (Thermo Fisher Scientific 65-0866-14). Together with the appropriate FMO controls and compensation bead set up, all mixtures were performed at 4°C in the dark for 20 min. Samples were fixed with 1X RBC lysis and fixation buffer (Biolegend #420302) for 15 min, then washed with cell staining buffer. Samples were resuspended in cold cell staining buffer and transferred through 30-μm cell strainer into round bottom tubes ready for FACS analysis. All samples were analyzed with a BD Symphony A1. Sample analyses using FlowJo software were based on 200,000 events captured.

Veracyte Brightplex technology: Sequential multiplex Immunohistochemistry (IHC)

Haematoxilin and eosin (H&E) staining was performed on 4μm-thick formalin-fixed paraffin-embedded (FFPE) tissue sections for a preliminary tissue evaluation. Slides were scanned with the NanoZoomer-XR (Hamamatsu) to generate digital images (20x). A pathologist identified the tumor area and provided qualitative assessment. 3 multiplex IHC panels were used: MDSC Panel, T Cells Effector Panel, Macrophage Panel.

Sequential multiplex IHC were carried out on 4μm-thick unstained sections with a Leica Bond RX autostainer (Leica Biosystems). Slides were deparaffinized and rehydrated in the autostainer according to the manufacturer's instructions. Antigen retrieval was performed with Bond Epitope Retrieval Solution #2 (Leica Biosystems), equivalent to EDTA pH 9.0, for detection of all biomarkers. Successive stainings were performed on the same FFPE slide.

The primary antibodies used for the MDSC Panel were anti-LOX1 (R&D Systems, catalog #MABS186), anti-CD15 (BD Biosciences, catalog #555400), anti-CD11B (Cell Signaling/Ozyme, catalog #49420S), anti-CD14 (Cell Marque/Sigma, catalog #114R-15), anti-S100A9 (Origene, catalog #UM800066) and anti-HLA-DR (Santa Cruz Biotech, catalog #Sc-53319). The primary antibodies used

for the T cell Effector Panel were anti-CD3 (HDX, NA), anti-CD8 (HDX, NA), anti-Ki67 (Cell Signaling Ozyme, catalog #9027), anti-GranzymeB (Dako, catalog #M72350), anti-PD-1 (Origène, catalog #UM800091), anti-PD-L1 (HDX, NA) and anti-CD11B (Cell Signaling, catalog #49420S). The primary antibodies used for the Macrophage Panel were anti-CD68 (Abcam, catalog #ab213363), anti-CD163 (Abcam, catalog #Ab182422), anti-CD11B (Cell Signaling, catalog #49420S), anti-CD15 (BD Biosciences, catalog #555400), anti-CD64 (Abcam, catalog #ab140779), anti-CD163 (Abcam, catalog #ab182422), anti-LOX1 (R&D Systems, catalog #MABS186) and anti-CD206 (R&D Systems, catalog #MAB25341).

Antibodies were diluted in Emerald antibody diluent (ESBE Scientifique; catalog #CMQ-936B09). The primary antibodies were detected using MACH 2 rabbit HRP polymer (Biocare, RHRP520L) or MACH 4 Universal HRP polymer as secondary antibody (Biocare, M4U534L). The labeling was visualized using aminoethyl carbazole carbazole (AEC Peroxidase Substrate Kit, Biocare, catalog #SK-4200; ImmPACTAMEC Red, Vector Lab, catalog #SK-4285) and hematoxylin counterstaining.

A human tonsil specimen was used as control for all immune biomarker's detection using qualitative acceptance criteria; specificity, staining location (nucleus/membrane), cell type, and lack of background or unspecific staining. After each individual staining, coverslipping was performed automatically by the workstation CTM6 with aqueous mounting (VectaMount AQ, VECTOR Laboratories, catalog #H-5501). The slides were digitalized in a NanoZoomer-XR scanner (Hamamatsu; 20x) and a visual quality control carried out. Between each staining cycle of the sequential multiplex, the labeling was eliminated by incubating the samples in ethanol, and the antibody complexes were denatured using a denaturing buffer.

Veracyte Brightplex technology: Digital *pathology analysis*

Each biopsy was analyzed using the Veracyte Digital Pathology Platform. Images obtained following sequential multiplex IHC workflow were aligned using the Veracyte Digital Pathology Platform. A pseudocolor image containing the information for the expression of all biomarkers was created. The latter was analyzed by HALO software (Indica Labs) for the identification of tumor areas using annotation tools. Next, positively stained cells were detected and quantified in the selected regions of interest using HALO software (Indica Labs). Phenotypes of interest were visually verified according to expected staining and quantified with Brightplex MultiplexR (HalioDx software). The final data were expressed as density (cells/mm^2) in the analyzed tumor regions.

NanoString analysis

RNA was extracted from FFPE tissues using QIAGEN RNeasy FFPE extraction kits (QIAGEN GmbH, Hilden, Germany). Annotations from the pathologist performing H&E staining were used to guide removal of normal tissue from the slides by spectrophotometry prior to nucleic acid extraction, which occurred after tissue deparaffinization and lysis. Each extracted RNA was independently quantified using a NanoDrop spectrophotometer (NanoDrop Technologies, Oxfordshire, UK) and quality checked (Agilent Bioanalyzer, Santa Clara, United States). Degradation assessment was quantified as the percentage of RNA fragments smaller than 300 base pairs using RNA 6000 Nano Kit (Agilent Bioanalyzer, Santa Clara, United States). Good sample quality was defined as less than 50% of RNA fragments of 50–300 base pairs in size.

RNA expression profiling was performed using the nCounter PanCancer IO360 Profiling Panel from NanoString (NanoString Technologies, Seattle, USA). The PanCancer IO360 Profiling Panel contains 770 probes. Hybridization was performed according to manufacturer's instructions. Hybridized probes were then purified and immobilized on a streptavidin-coated cartridge using the nCounter Prep Station (NanoString Technologies). Data collection was carried out on the nCounter Digital Analyzer (NanoString Technologies) following the manufacturer's instructions to count individual fluorescent barcodes and quantify target RNA molecules present in each sample. For each assay, a scan of 490 fields of view was performed.

Raw data from the NanoString nCounter were processed using NanoString recommendation. The quality control enable to keep good quality data with a binding density that range between 0.05 and 2.25. The linearity of positive controls was checked using the R2 of regression between the counts and the concentration of positive controls. No samples were removed from analysis based on a threshold of $R2 < 0.75$.

For normalisation of raw data by nCounter normalisation for immunosign-21 only, the background was removed using thresholding method at the mean +2 standard deviation of negative controls. Note sample 0107-0001 patient had low absolute counts so was excluded from the normalisation process. The counts were then normalized using positive normalization factor. Samples showing positive normalization factors out of the range 0.3–3 were removed from the analysis. A second normalization was performed using Housekeeping gene normalization factor. Only the most stable housekeeping genes were selected for this normalization step using the Variance vs. Mean relationship. One sample showed a housekeepering normalization factor out of the range 0.1–10 so was flagged and removed in certain analyses as specified. Gene expression from Immunosign signatures (Immunosign 15 and Immunosign 21) were assessed as described previously.[23]

Differential expression analyses

Raw counts of nCounter Nanostring PanCancer IO panel were extracted for 24 patients for Screening and C2D16 timepoints. Unwanted variation was determined by specifying the following housekeeping genes as controlGenes within the DESeq2 v1.36.0 estimateSizeFactors function and as negative control genes within RUVSeq v1.30.0 function RUVg: "UBB", "PUM1", "TLK2", "DNAJC14", "SF3A1", "POLR2A″, "SDHA", "STK11IP", "NRDE2", "TBP", "OAZ1", "TBC1D10B″, "GUSB", "PSMC4", "TMUB2", "MRPL19", "G6PD","ERCC3". These factors were included in both data normalisation and transformation by Variance

Stabilising transformation (VST) for data visualisation, and also for differential gene expression by DESeq2.[54,61,63,64] The screening sample for patient, 0107-0001, had low absolute counts but clustered normally after unwanted variation processing and DESeq2 analysis so was included in analyses.

Genes were determined statistically significant by Wald statistic with threshold adjusted p value <0.05 and log2FoldChange > +/− 0.5. Differential gene expression was subjected to logfold change shrinkage for visualisation by EnhancedVolcano v1.14.0. Pheatmap v1.0.12 was used to visualise VST gene expression. Rstatix for statistical testing v0.7.1. Package fgsea v1.22.0 was used to determine gene set enrichment, specifying pathways either created or identified from the literature. For input into fast gene set enrichment (fgsea), genes were ranked based on DESeq2 wald statistic and determined statistically significant based on adjusted p value of 0.05. R version v4.2.0 was used for this analysis. p values were adjusted for based on multiple testing with the rest of the gene sets tested. Gene pathways are detailed in Table S8.

For gene expression biomarker signature, the VST counts of genes significantly differentially expressed (Padj <0.1) between patients with progressive disease and non-progressive disease were extracted and a signature determined by subtracting the sum of the gene expression counts downregulated by responders (“VTCN1”, “CCNE1”, “MARCO”, “WNT10A”, “EDN1”, “ANLN”) from the sum of those upregulated in responders (“HDC”, “IL1B”, “ARG2”, “ACVR1C″, “NCAM1”, “CASP9”, “CST2”, “WNT5A″, "DLL4″). This method is similar to those described previously.[30,64–66]

OLINK

Pre-treatment plasma samples from TIME POINT and OUTREACH patients were sent to OLINK at the Immunogenomics Facility in Leeds for quantification of 92 proteins within the OLINK Target 96 Inflammation panel by proximity extension assay. The quality of each sample was determined based on a threshold of variation of two internal controls, the detection control and incubation control. Samples were included in analyses only if they passed the quality control thresholds. Protein quantification was provided as normalised protein expression units (NPX). Statistical analysis between patient clinical response groups was performed by an unpaired two-tailed wilcox in R v4.2.0 using rstatix and EnhancedVolcano. A combined OLINK signature was determined for each of the TIME POINT patients by taking the value of protein, DNER from the sum of IL15RA, t_4EBP1, TNF, CSF1 and IL2RB.

Protein detection of CEBPA in monocytes and lymphocytes

Blood collected in a Vacutest K2EDTA tube was diluted 1:3 in PBS, carefully overlayed on top of half volume of Ficoll Plaque Plus (GE Healthcare, Cat. No. GE17-1440-02), and then centrifuged in a swinging bucket centrifuge for 30 min at 400g, RT, without break. After centrifugation, the middle layer containing peripheral blood mononuclear cells (PBMC) was collected, washed with PBS, and spun down. The cell pellet was resuspended in X-Vivo 15 media (Lonza, Cat. No. BE02-060F), carefully overlayed on top of 3 times the volume of Percoll working solution (Fisher Scientific Cat. No. 11500744), and then centrifuged at 580xg for 15 min at RT on a swinging bucket centrifuge. Monocytes enriched fraction is recovered from the top layer, while the lymphocytes enriched fraction from the bottom one as described previously.[67] Population enrichment was assessed by staining healthy blood following the StainExpress Immune Cell Composition Cocktail kit (Miltenyi Cat. No. 130-127-637) protocol. On average monocyte enrichment was 50%, while lymphocyte enrichment was 61%.

Pellets are washed with PBS supplemented with 1mM EDTA and then lysed in RIPA buffer supplemented with Halt Protease and Phosphatase Inhibitor Cocktails 100X (Fisher Scientific, Cat. No. 78440). The protein lysates were sonicated and centrifuged for 15 min at high speed at 4°C. DC Protein Assay Kit I, (Bio-Rad cat. No. 5000111) was used to measure the concentrations of the proteins in the supernatant. Samples were prepared as per vendor’s protocol. Automated capillary immunoassay (Simple Western) was performed on a Jess system (Protein Simple, San Jose, CA, USA). Analyses were performed on the 12–230 kDa Separation Module (SM-W004) according to the manufacturer’s instructions. The target proteins were immune-probed with anti-CEBPA primary antibody (D56F10 XP Rabbit mAb Cat No 8178, Cell Signaling Technology) diluted in antibody diluent (Protein Simple 042–203). Samples and reagents were loaded onto the plate and run on the Jess machine. At the end of the run, the electropherograms, automatically generated for both fluorescent standards and samples, were evaluated in each capillary for proper peak assignation and when required, manual correction was applied. Within-capillary total protein normalization was performed to account for any differences in protein loading. Jess operations and peak calculations were performed using the Compass software (ProteinSimple). Relative protein amounts were assessed using the corrected area of chemiluminescent peaks.

QUANTIFICATION AND STATISTICAL ANALYSIS

Statistical analysis

Unless specified, no patients were excluded from analyses. Descriptive statistics were used to characterise safety analyses. Sample sizes for each dose were determined on the basis of observed toxicities, not statistical considerations. Plasma CEBPA-51 concentrations over the first dosing interval, after once-weekly dosing with MTL-CEBPA, were used to derive non-compartmental PK parameters using Phoenix WinNonlin version 7.0 (Certara). Statistical analyses were performed with GraphPad Prism 9 (version 9.1.2 (225)) for macOS (GraphPad Software, Inc., San Diego, CA) or using rstatix and base R v4.2.0. As specified in each figure legend, unpaired t-test with Welch’s correction or false discovery rate adjustment or Kruskal-Wallis test was used to analyze groups, and paired wilcox tests when samples were paired by timepoint. Differences were considered significant at a p value of <0.05. The

definition of clinical response is detailed in the figure legends but unless stated, refers to clinical response by RECIST or if unavailable, at C2 D22 determined by site. Kaplan-Meier methodology using the survival and survminer packages were used to report median and 95% confidence intervals (CI) for progression-free survival (PFS) with significance determined by log-rank test. Progression-free survival (PFS) is defined as the time from the date of first treatment, to the time of progression. PFS was censored based on radiological progression as defined by RECIST. Univariable and multivariable cox regression analyses were performed in R using the survival and survminer packages.

ADDITIONAL RESOURCES

This study was registered with ClinicalTrials.gov, number NCT04105335.

Cell Reports Medicine, Volume *6*

Supplemental information

TIMEPOINT, a phase 1 study combining MTL-CEBPA with pembrolizumab, supports the immunomodulatory effect of MTL-CEBPA in solid tumors

Ruth Plummer, Mikael H. Sodergren, Rose Hodgson, Bríd M. Ryan, Nina Raulf, Joanna P. Nicholls, Vikash Reebye, Jon Voutila, Laura Sinigaglia, Tim Meyer, David J. Pinato, Debashis Sarker, Bristi Basu, Sarah Blagden, Natalie Cook, Thomas R. Jeffrey Evans, Jeffrey Yachnin, Cheng E. Chee, Daneng Li, Anthony El-Khoueiry, Maria Diab, Kai-Wen Huang, Madhava Pai, Duncan Spalding, Thomas Talbot, Marcus S. Noel, Bridget Keenan, Devalingam Mahalingam, Min-Sun Song, Mélanie Grosso, Denis Arnaud, Aurelie Auguste, Dimitris Zacharoulis, Jan Storkholm, Iain McNeish, Robert Habib, John J. Rossi, and Nagy A. Habib

A

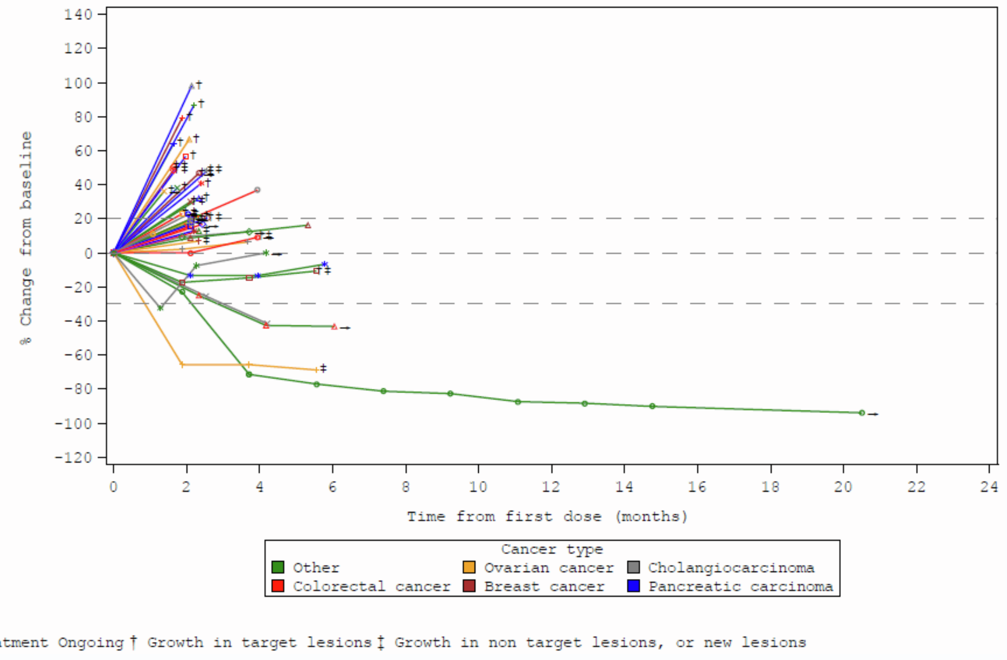

Supplementary Figure 1. Best objective tumour response by patient across treatment groups in TIMEPOINT clinical trial.

A. Spider plot waterfall plot illustrating patient best objective tumour response across treatment groups in Phase 1a and Phase 1b. Note: an i prefix represents a response evaluated according to the iRECIST or irRECIST criteria. Patients with no post-baseline RECIST assessments are excluded from this figure. iRECIST=immune RECIST; irRECIST=immune-related RECIST; PD=progressive disease; PR=partial response; SD=stable disease; UPD=unconfirmed progressive disease. Each line represents one patient. Related to Figure 1.

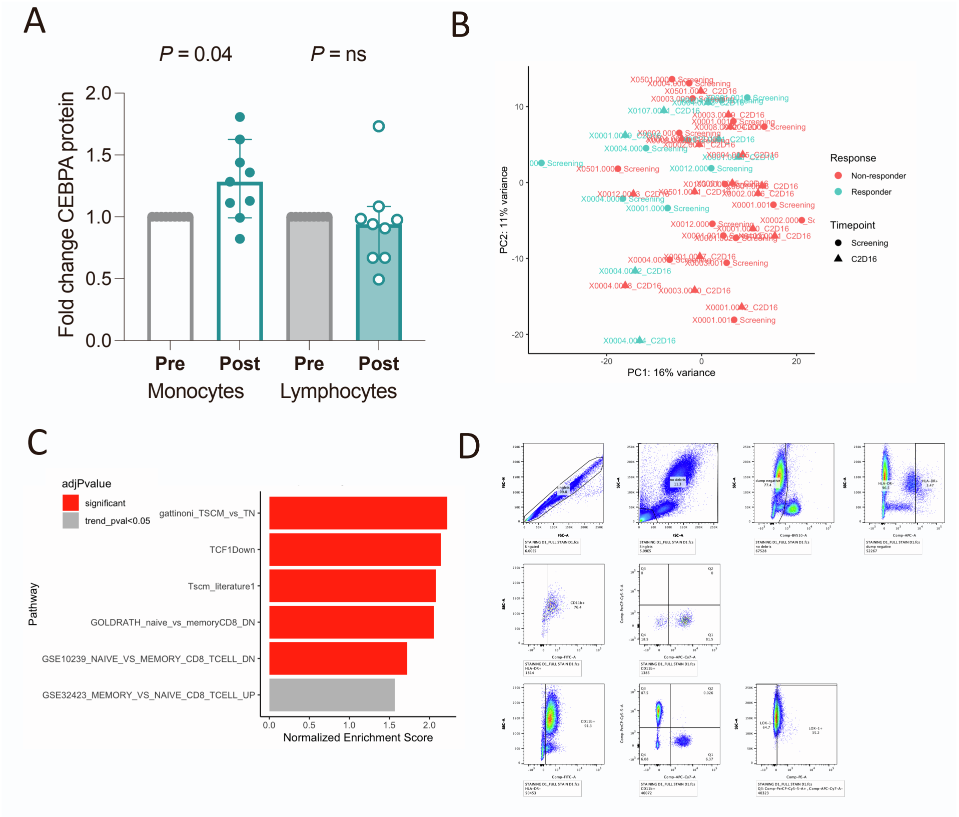

Supplemental figure 2. Pharmacodynamic changes seen in patient tumour and peripheral blood samples post-treatment with MTL-CEBPA in combination with pembrolizumab.

A. Detection of CEBPA protein in patient monocyte or lymphocyte fraction enriched from peripheral blood by density gradient centrifugation, pre (C1D1) and post treatment (C1 D2) with MTL-CEBPA. Each point represents one patient. Bar is at median with 95% confidence intervals as error bars. Significance was determined by one sample Wilcoxon test with hypothetical median value of 1, P values are unadjusted. B. PCAplot of 48 patient samples of Nanostring transcriptomic data analysed with DESeq and VST normalization and stabilization for batch effects using pre-assigned housekeepers. Note sample 0107-0001 patient had low absolute counts but clusters normally with DESeq2 analysis. Response refers to clinical response at C2 D22 determined by site. C. FGSEA analysis of differentially expressed gene pathways between pre- and post-treatment timepoints across all patients, 14 using T stem cell memory gene sets identified or curated from literature (Supplementary table 8). Pathways in red reached significance (P adjust < 0.05), grey pathways were significant pre-adjustment for multiple testing (P <0.05). D. Representative flow cytometry plots of full gating path example of PMN-MDSCs from peripheral blood. Gating is based on FMOs for each marker. Related to Figure 2.

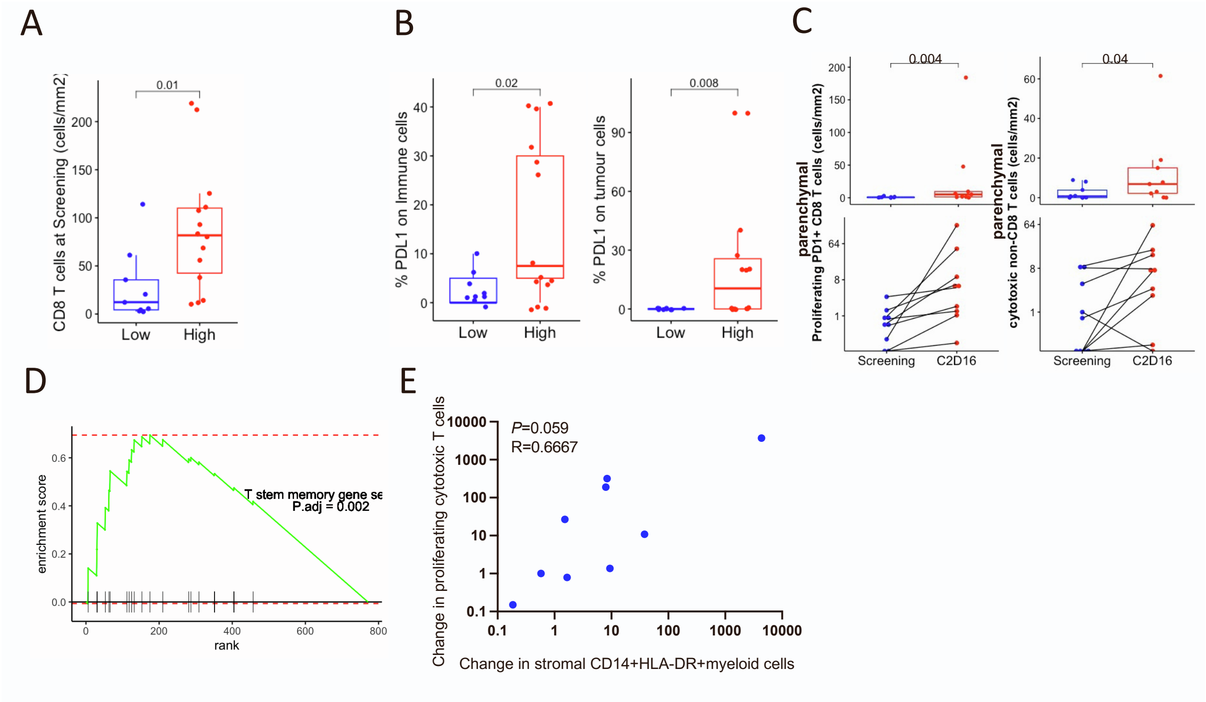

Supplementary figure 3. Patient tumour biopsies have different levels of immune infiltration before treatment, with patients that have lower numbers of immune cells at baseline demonstrating increased intratumoural immune infiltration following treatment with MTL-CEBPA in combination with pembrolizumab.

A. IHC quantification of CD3+CD8+ T cells at pre-treatment (Screening) between of 9 tumour biopsies with an immune desert TME (IS21 < 10) and 14 patient biopsies with an immune-inflamed TME (IS21 > 10). The threshold of 10 for IS21 was arbitrarily chosen as it is the midpoint of the assay readout, which gives values between 0-20. P value is determined by two-sided Wilcoxon test. B. IHC quantification of PDL1 presence on immune cells (IC: left) or tumour cells (TPS: right) at pre-treatment (Screening) between of 9 tumour biopsies with an immune desert TME (IS21 < 10) and 14 patient biopsies with an immune-inflamed TME (IS21 > 10). P value is determined by two-sided Wilcoxon test. C. IHC quantification of parenchymal CD3+ CD8+KI67+GrzB+PD1+ T cells (left) and parenchymal CD3+CD8-GZMB+ T cells (right) between pre- (Screening) and post-treatment (C2D16) of 9 paired biopsies with a immune desert TME (IS21 < 10). P value is determined by Paired Two-sided Wilcoxon test. For A, B and C, the box plots show the data distribution, where the center line denotes the median, the box edges show the interquartile range each point is one patient. D. Gene set enrichment plot of T stem memory gene set, “GSE10239_NAIVE_VS_MEMORY_CD8_TCELL_DN”. Significance was determined by fgsea and adjusted for multiple testing based on 14 gene sets tested (Supplementary table 8). E. Pearson correlation of change in stromal CD14+HLA-DR+ myeloid cells from

Screening to C2D16 with change in proliferating cytotoxic CD8 T cells in patients that had cold TME at baseline (N=9). Related to figure 3.

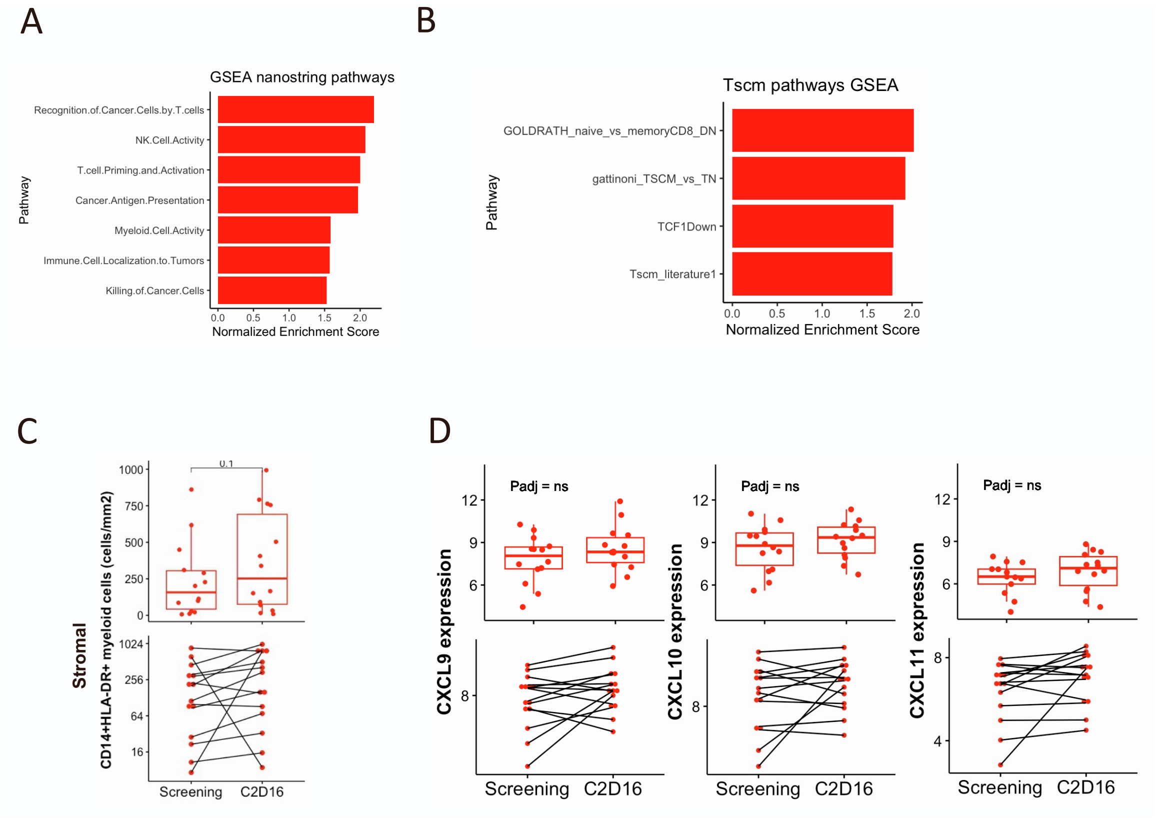

Supplementary figure 4. Patients with high levels of immune activity at baseline demonstrate modest immune infiltration following combination treatment with MTL-CEBPA in combination with pembrolizumab.

A. FGSEA analysis of differentially expressed genes between pre- (Screening) and post-treatment (C2D16) of 14 paired biopsies with an immune inflamed TME (IS21 > 10), using supplied Nanostring pathways of the IO Pan-cancer panel or B. T stem cell memory pathways. Pathways in red reached significance with adjustment for multiple testing for 13 (A) or 14 (B) gene sets (P adjust < 0.05). C. IHC quantification of stromal CD11b+CD14+CD15-HLA-DR+ cells between pre- (Screening) and post-treatment (C2D16) in the stroma of 14 paired biopsies with an immune inflamed TME (IS21 > 10). P value is determined by Paired Two-sided Wilcoxon test. D. Quantification of CXCL genes post-treatment in immune inflamed tumours by nanostring. Data are VST values normalised for batch with housekeepers, with significance determined by DESeq2 on raw data. The box plots in C and D show the data distribution, where the center line denotes the median, the box edges show the interquartile range and each point represents one patient. Related to Figure 3.

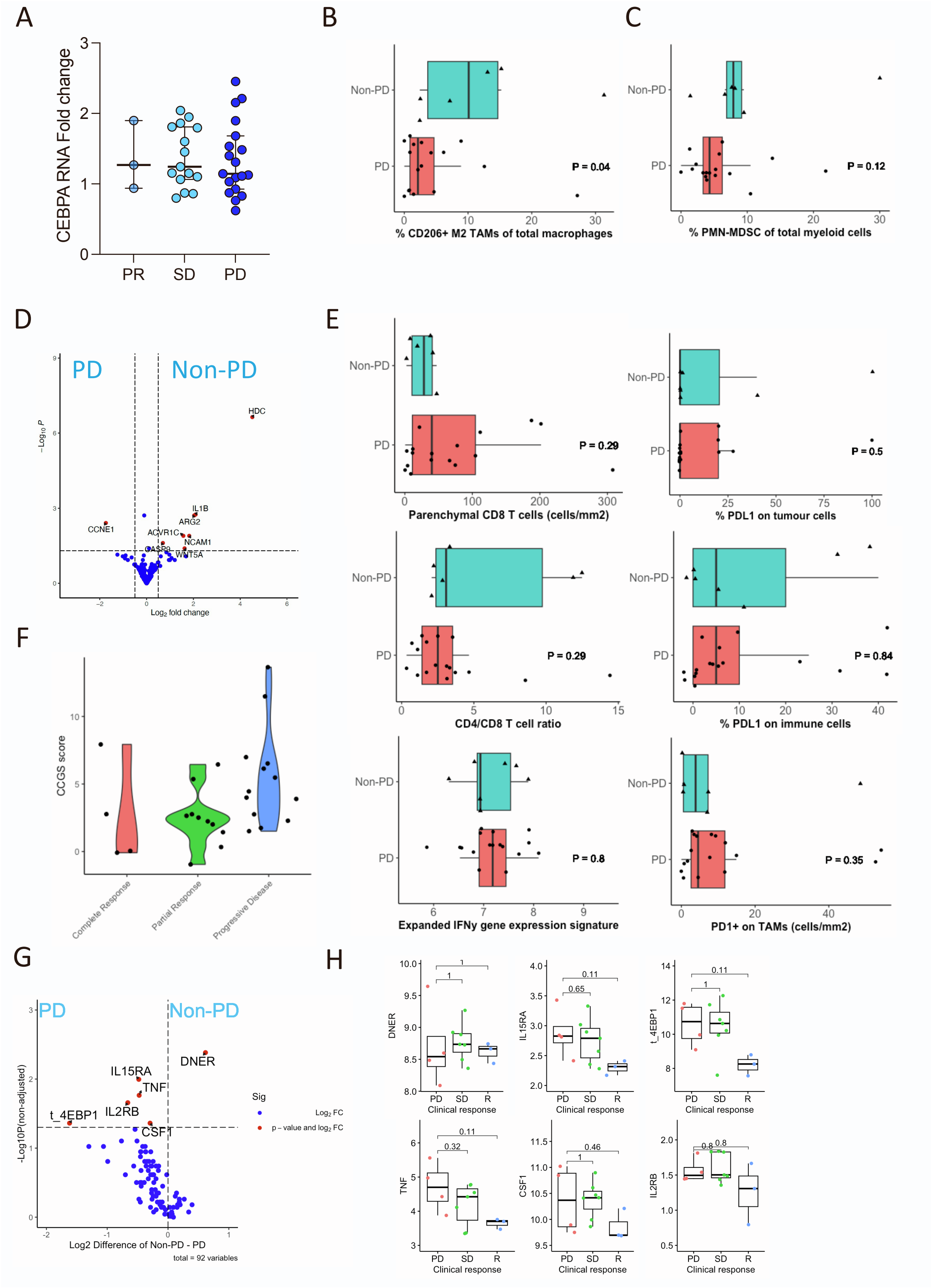

Supplementary Figure 5. Patients that have signals associated with myeloid-derived immunosuppression pre-treatment are more likely to demonstrate disease stabilisation following treatment with MTL-CEBPA in combination with pembrolizumab.

A. Fold change of CEBPA RNA at C1 D2 compared to pre-treatment, C1-D1 in TIMEPOINT in patients with PR, SD and PD. Response refers to clinical response at C2 D22 determined by site. Bar is at median with 95% confidence interval error bars, each point is one patient. Significance was tested with a Kruskal-Wallis test, with no significance reached at a P value of P = 0.05. B. Visualisation of CD11b+CD68+CD64-CD206+CD163- cells (CD206+ M2 TAMs) as a proportion of total macrophages (CD11b+CD68+ cells) between patients with PD and non-PD. C. Visualisation of CD11b+CD15+CD14-HLA-DR-LOX1+ (PMN-MDSC) cells as a proportion of total myeloid cells (CD11b+ cells) between patients with PD and non-PD. For B and C, each point is one patient, significance values were determined with a Two-sided Wilcoxon test. E. Visualisation of previously identified pembrolizumab monotherapy biomarkers between patients with PD and non-PD. Significance was determine using Two-sided Wilcoxon tests for each plot independently without adjustment for multiple testing. F. CCGS determined by RNASeq of pre-treatment gene expression in pembrolizumab treated melanoma patients from publicly available dataset, GSE78220, each point is one patient. G. Volcano plot of OLINK serum analysis of circulating proteins using Inflammation panel of patient serum at baseline (pre-treatment) in TIMEPOINT between patients with different responses. Significance was determined with an unpaired two-tailed Wilcoxon test, using patients with PD as the control group. P values are non-adjusted. All genes are labelled. Each point is one patient. For B-E and G, response refers to clinical response at C2 D22 determined by site. H. OLINK serum analysis of circulating proteins using Inflammation panel of patient serum at baseline (pre-treatment) in OUTREACH clinical trial as NPX values. Response refers to PD or non-PD clinical response determined by RECIST. Significant was determined with an unpaired two-tailed Wilcoxon. P values are non-adjusted. The box plots in B, C, E, and H show the data distribution, where the center line denotes the median, the box edges show the interquartile range and each point is one patient. Related to Figures 4 and 5.

Supplementary tables

Supplementary Table 1. Study population analysis sets by tumour type recruited in TIMEPOINT clinical trial.

EFR=evaluable for response; PKAS=pharmacokinetic analysis set; SAF=safety analysis set.

'Other' category contains the following cancer types: epithelioid mesothelioma, thymic cancer metastatic, hepatocellular carcinoma, eccrine carcinoma, adenocarcinoma, lung neoplasm malignant (term amended to mesothelioma after the data cut-off for this interim analysis), extrahepatic cholangiocarcinoma (in 2 patients), neuroendocrine tumour, leiomyosarcoma, malignant peritoneal neoplasm, anal squamous cell carcinoma, and mesothelioma. Related to Figure 1, table 1 and Supplementary figure 1.

**Cancer type**	**Breast cancer**	**Intrahepatic Cholangiocarcinoma**	**Colorectal cancer**	**Ovarian cancer**	**Pancreatic carcinoma**	**Other**	**Overall**
**Patient number**	**(N=4)**	**(N=7)**	**(N=9)**	**(N=8)**	**(N=9)**	**(N=13)**	**(N=50)**
**SAF**	4 (100.0)	7 (100.0)	9 (100.0)	8 (100.0)	9 (100.0)	13 (100.0)	50 100.0)
**PKAS**	1 (25.0)	1 (14.3)	1 (11.1)	4 (50.0)	3 (33.3)	6 (46.2)	16 (32.0)
**EFR**	3 (75.0)	5 (71.4)	6 (66.7)	7 (87.5)	8 (88.9)	11 (84.6)	40 (80.0)

Supplementary Table 2: Patient demographics at baseline in TIMEPOINT clinical trial.

QW=dosing frequency is once weekly, BMI= body mass index, BSA = body surface area, ECOG= Eastern Cooperative Oncology Group score. Related to Figure 1, table 1 and Supplementary figure 1.

	**Phase 1a**	**Phase 1a**	**Phase 1a**	**Phase 1b**	**MTL-CEBPA**
	**MTL-CEBPA**	**MTL-CEBPA**	**MTL-CEBPA**	**MTL-CEBPA**	**overall**
	**70 mg/m^2 QW**	**98 mg/m^2 QW**	**130 mg/m^2 QW**	**130 mg/m^2 QW**	**(N=50)**
	**(N=4)**	**(N=3)**	**(N=3)**	**(N=40)**	
**Age at time of informed consent (years)**					
Median (min;max)	44.5 (33;56)	56.0 (21;72)	48.0 (40;60)	62.5 (25;80)	58.5 (21;80)
**Gender**					
Female, n (%)	3 (75.0)	2 (66.7)	2 (66.7)	26 (65.0)	33 (66.0)
Male, n (%)	1 (25.0)	1 (33.3)	1 (33.3)	14 (35.0)	17 (34.0)
**Race**					
White/Caucasian, n (%)	4 (100.0)	1 (33.3)	2 (66.7)	30 (75.0)	37 (74.0)
Asian or Asian British, n (%)	0	2 (66.7)	1 (33.3)	5 (12.5)	8 (16.0)
Black or Black British, n (%)	0	0	0	2 (5.0)	2 (4.0)
Mixed	0	0	0	2 (5.0)	2 (4.0)
Other	0	0	0	1 (2.5)	1 (2.0)
**Baseline BMI (kg/m^2)**					
Mean ± StD	23.6 ± 3.2	24.4 ± 9.6	23.4 ± 3.5	25.2 ± 4.8	24.9 ± 4.8
Median (min;max)	22.9 (20.4;28.0)	19.4 (18.3;35.4)	22.0 (20.7;27.4)	24.4 (14.2;38.2)	24.0 (14.2;38.2)
**Baseline BSA (kg/m^2)**					
Median (min;max)	1.7 (1.6;1.7)	1.4 (1.3;1.8)	1.8 (1.76;1.78)	1.8 (1.4;2.2)	1.8 (1.3;2.2)
**ECOG status at baseline** Median (min;max)					
0, n (%)	3 (75.0)	2 (66.7)	1 (33.3)	17 (42.5)	23 (46.0)
1, n (%)	1 (25.0)	1 (33.3)	2 (66.7)	23 (57.5)	27 (54.0)

**Prior lines of chemotherapy**					
0	0	1 (33.3)	0	0	1 (2.0)
1	0	1 (33.3)	1 (33.3)	4 (10.0)	6 (12.0)
2	1 (25.0)	1 (33.3)	1 (33.3)	12 (30.0)	15 (30.0)
3	1 (25.0)	0	0	11 (27.5)	12 (24.0)
4	0	0	1 (33.3)	4 (10.0)	5 (10.0)
5	1 (25.0)	0	0	6 (15.0)	7 (14.0)
6	1 (25.0)	0	0	3 (7.5)	4 (8.0)

Supplementary table 3: Best objective tumour response across Treatment groups in Phase 1a and Phase 1b of TIMEPOINT clinical trial.

Note: an i prefix represents a response evaluated according to the iRECIST or irRECIST criteria. iRECIST=immune RECIST; irRECIST=immune-related RECIST; mRECIST=modified RECIST; PD=progressive disease; PR=partial response; QW=once a week; RECIST=Response Evaluation Criteria in Solid Tumours; SD=stable disease; trRECIST=tumour response RECIST; UPD=unconfirmed progressive disease. Related to Figure 1, table 1 and Supplementary figure 1.

	**Breast cancer** (N=3)	**Intrahepatic Cholangiocarcinoma** (N=5)	**Colorectal cancer** (N=6)	**Ovarian cancer** (N=7)	**Pancreatic carcinoma** (N=8)	**Other** (N=11)	**Overall** (N=40)
**Responder**	0	1 (20.0)	0	1 (14.3)	0	2 (18.2)	4 (10.0)
PR	0	1 (20.0)	0	1 (14.3)	0	2 (18.2)	4 (10.0)
PR (RECIST 1.1)	0	1 (20.0)	0	1 (14.3)	0	0	2 (5.0)
PR (mRECIST)	0	0	0	0	0	1 (9.1)	1 (2.5)
iPR (iRECIST)	0	0	0	0	0	1 (9.1)	1 (2.5)
**Non-responder**	3 (100.0)	4 (80.0)	6 (100.0)	6 (85.7)	8 (100.0)	9 (81.8)	36 (90.0)
SD (RECIST 1.1)	0	3 (60.0)	1 (16.7)	3 (42.9)	0	3 (27.3)	10 (25.0)
iSD (trRECIST)	0	0	1 (16.7)	0	0	0	1 (2.5)
PD (RECIST 1.1)	3 (100.0)	1 (20.0)	3 (50.0)	3 (42.9)	7 (87.5)	6 (54.5)	23 (57.5)
iUPD (iRECIST)	0	0	1 (16.7)	0	1 (12.5)	0	2 (5)

Supplementary table 4. Patient information for those with tissues analysed by biological assays for exploratory analysis. * For this patient, only pre-treatment tumour biopsy sample was available. Related to figures 2-5 and supplemental figures 2-5.

Patient ID	Tumourtype/Aetiology	IHC T cells	IHC myeloid	IHC PDL1	Nanostring	PDL1	OLINK
0001.0009	EccrineAdenocarcinoma	x	x	x	x	x	x
0001.0011	Oesophago-gastricAdenocarcinoma	x	x	x	x	x	x
0001.0012	OvarianSerousCarcinoma	x	x	x	x	x	x
0001.0015	Recto-sigmoid	x	x	x	x	x	x
0001.0016	Ovarian	x	x	x	x	x	x
0001.0017	ExtrahepaticCholangiocarcinoma	x	x	x	x	x	x
0001.0020	Colon	x	x	x	x	x	x
0002.0001	Pancreatic	x	x	x	x	x	
0002.0006	BreastCancer(TripleNeg)	x	x	x	x	x	x
0003.0009	Ovarian(lowgradeserous)	x	x	x	x	x	x
0003.0010	Ovarian(HighgradeSerous)	x	x	x	x	x	x
0004.0002	FibrolamellarHCC	x	x	x	x	x	x
0004.0003	Colon	x	x	x	x	x	x
0004.0004	LungMetastaticAtypicalLung	x	x	x	x	x	x
0004.0005	Pancreatic	x	x	x	x	x	x
0004.0006	Recto-sigmoid	x	x	x	x	x	x
0004.0008	Sarcoma	x	x	x	x	x	
0008.0002	PancreaticAdenocarcinoma	x	x	x	x	x	x
0012.0001	IntrahepaticCholangiocarcinoma	x	x	x	x	x	x
0012.0003	IntrahepaticCholangiocarcinoma	x	x	x	x	x	x
0103.0001	Pancreatic	x	x	x	x	x	x
0107.0001	RectalCancer				x*		x
0501.0001	IntrahepaticCholangiocarcinoma	x	x	x	x	x	x

0501.0002	Breast	x	x	x	x	x	x
0001.0013	Ovarian						
0001.0014	Mesothelioma(Epitheloid)						
0001.0003	Breast(TripleNeg)						
0001.0004	Mesothelioma(Epitheloid)						x
0001.0005	Ovarian						x
0001.0006	Thymic						
0001.0007	Fibrolamellar						
0001.0018	IntrahepaticCholangiocarcinoma						x
0001.0019	Rectal						
0101.0002	Pancreatic						
0101.0004	IntrahepaticCholangiocarcinoma						x
0001.0021	Mesothelioma(Epitheloid)						
0005.0002	Ovarian(Peritoneal)Adenocarcinoma						
0010.0002	Pancreatic(Neuroendocrine)						
0002.0002	PancreaticMetastaticAdenocarcinoma						
0002.0003	BreastCancer						
0003.0002	Colorectal						
0003.0004	intrahepaticCholangiocarcinoma						
0003.0005	Ovarian						
0003.0006	ExtrahepaticCholangiocarcinoma						
0003.0008	PancreaticAdenocarcinoma						
0101.0006	Pancreatic						
0101.0007	ColonCancer						
0005.0001	Rectal						x
0103.0003	IntrahepaticCholangiocarcinoma						x
0501.0003	Mesothelioma(Peritoneal)						x
0101.0005	Rectal(anal)Cancer						

0201.0001	Ovariancancer-highgradeserous						
